# Author Correction: Deletion and tandem duplications of biosynthetic genes drive the diversity of triterpenoids in *Aralia elata*

**DOI:** 10.1038/s41467-022-30370-z

**Published:** 2022-05-06

**Authors:** Yu Wang, He Zhang, Hyok Chol Ri, Zeyu An, Xin Wang, Jia-Nan Zhou, Dongran Zheng, Hao Wu, Pengchao Wang, Jianfei Yang, Ding-Kun Liu, Diyang Zhang, Wen-Chieh Tsai, Zheyong Xue, Zhichao Xu, Peng Zhang, Zhong-Jian Liu, Hailong Shen, Yuhua Li

**Affiliations:** 1 State Key Laboratory of Tree Genetics and Breeding, Northeast Forestry University, Harbin, 150040 China; 2 College of Life Sciences, Northeast Forestry University, Harbin, 150040 China; 3 Key Laboratory of Saline-alkali Vegetation Ecology Restoration (Northeast Forestry University), Ministry of Education, Harbin, 150040 China; 4 Heilongjiang Key Laboratory of Plant Bioactive Substance Biosynthesis and Utilization, Northeast Forestry University, Harbin, 150040 China; 5 School of Forestry, Northeast Forestry University, Harbin, 150040 China; 6 Key Laboratory of Orchid Conservation and Utilization of National Forestry and Grassland Administration at College of Landscape Architecture, Fujian Agriculture and Forestry University, Fuzhou, 350002 China; 7 Orchid Research and Development Center, National Cheng Kung University, Tainan, 701 Taiwan China; 8 Department of Life Sciences, National Cheng Kung University, Tainan, 701 Taiwan China; 9 Present Address: Biochemistry Institute, University of Science, Pyongyang, 999093 Democratic People’s Republic of Korea

**Keywords:** Secondary metabolism, Biocatalysis, Genome evolution, Metabolic engineering

Correction to: *Nature Communications* 10.1038/s41467-022-29908-y, published online 25 April 2022.

In the original version of this Article, the label of “*DDS* loss” in Fig. 8a was shifted under the label “Araliaceae-common WGD”. In addition, in the same figure panel, the labels of “Dc WGD-α” and “Dc WGD-β” were shifted toward the label “Apiales ancestor” (see incorrect figure below).



These have been corrected in both the PDF and HTML versions of the Article (see corrected figure below).

