# Supplementary material for: Deletion and tandem duplications of biosynthetic genes drive the diversity of triterpenoids in Aralia elata
Source: Nat Commun. 2022 Apr 25;13:2224. doi: 10.1038/s41467-022-29908-y (PMC9038795; doi:10.1038/s41467-022-29908-y)
Supplement: Supplementary file 1 — Supplementary Information [file 41467_2022_29908_MOESM1_ESM.pdf]

Deletion and tandem duplications of biosynthetic genes drive the diversity of triterpenoids in *Aralia elata*

Wang *et al*.

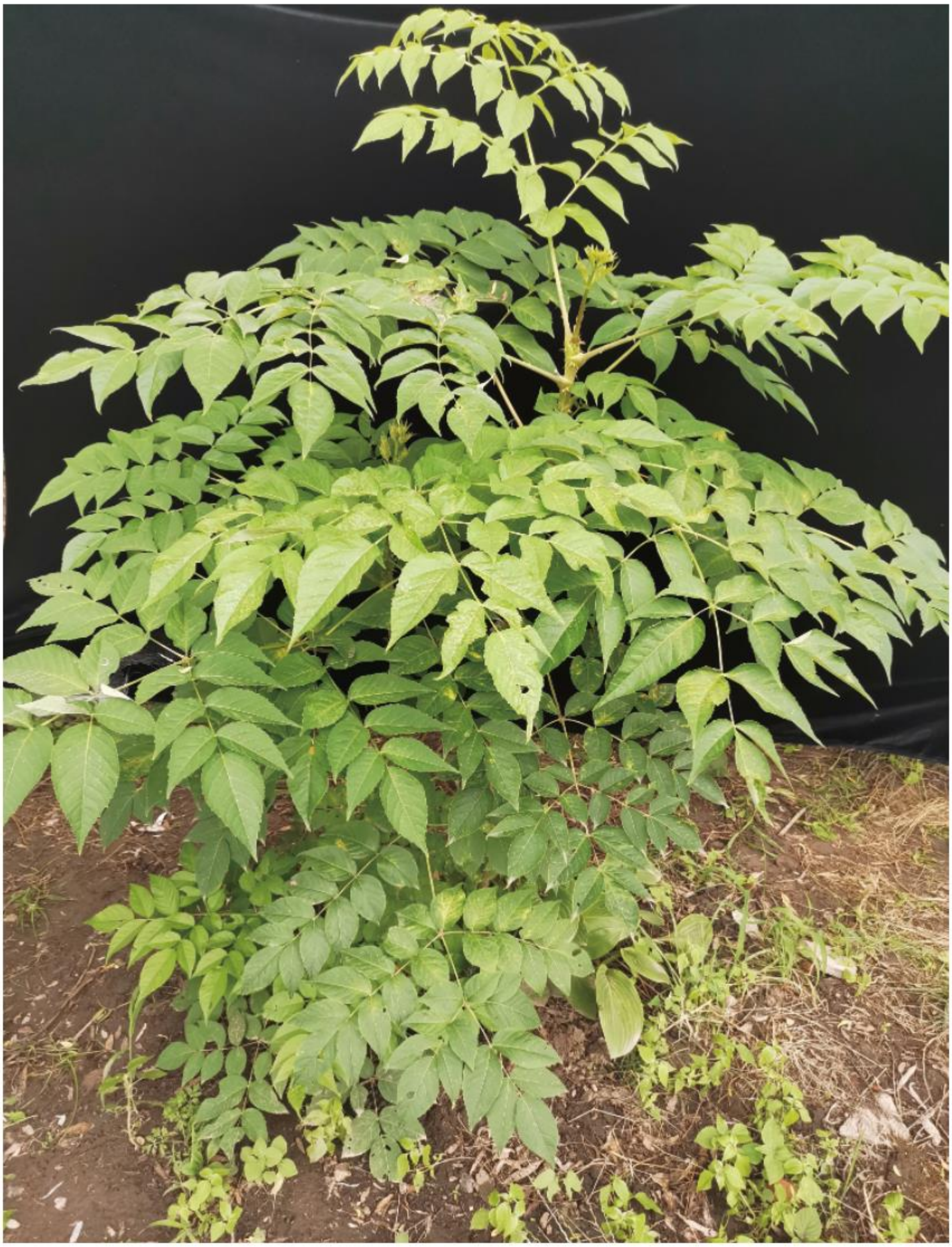

Supplementary Figure 1. ***Aralia elata*****, growing in Harbin, Heilongjiang Province, northeast China.**

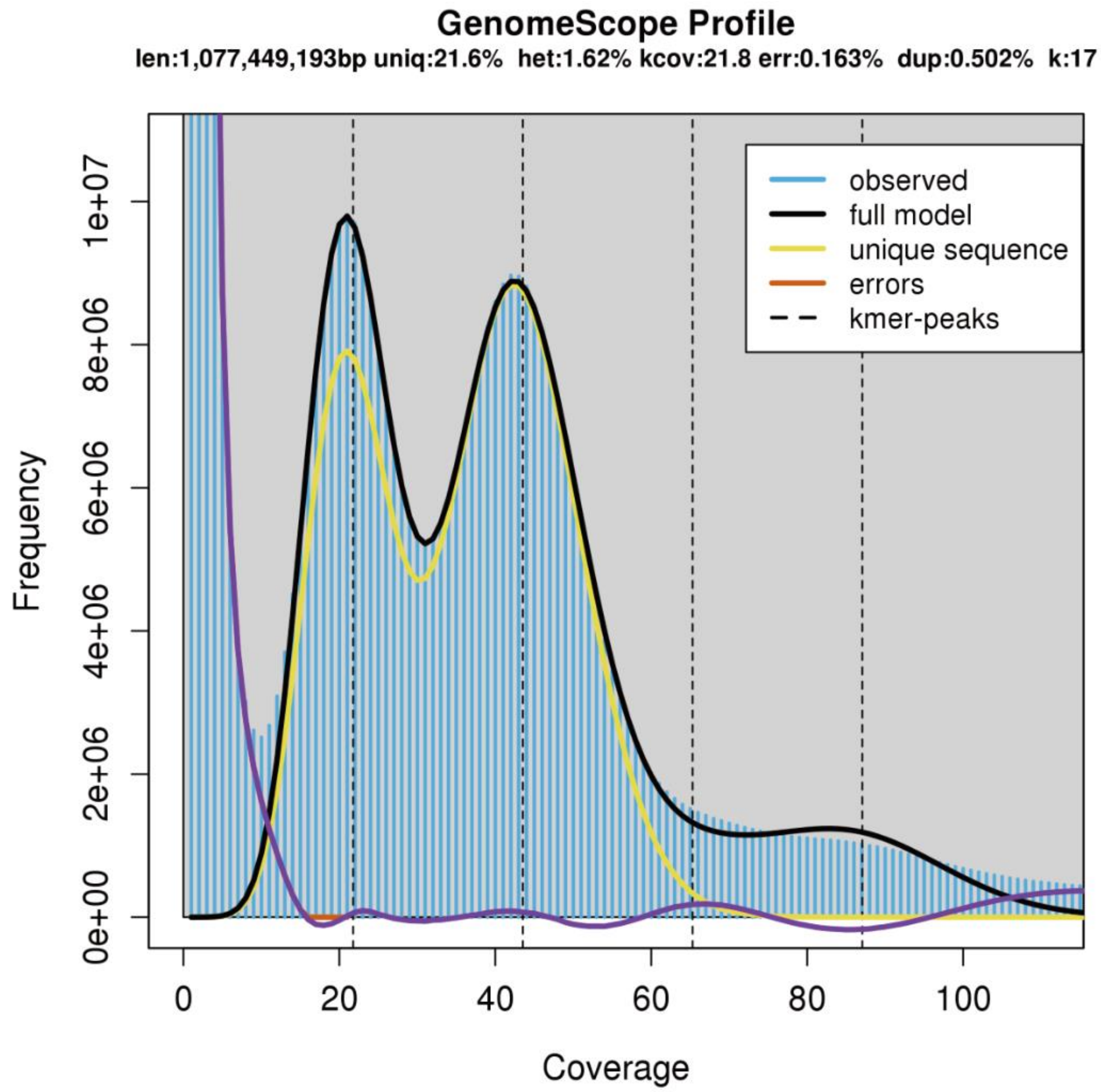

Supplementary Figure 2. Genome size and heterozygosity of *A. elata* estimated using 17 *K*-mer distribution. The paired-end reads (150 bp) from short insert-size libraries (300-500 bp) were used to generate the 17-mer frequency curve. The horizontal axis represented the *K*-mer depth, namely the number of times occurred. The volume of *K*-mers was plotted against the frequency at which they occur. According to the distribution, we estimated the genome size of *A. elata* to be 1.08 Gb, with a 1.62% heterozygosity rate and a 78.38 % repeat sequence, based an analysis of *K*-mer numbers/depths.

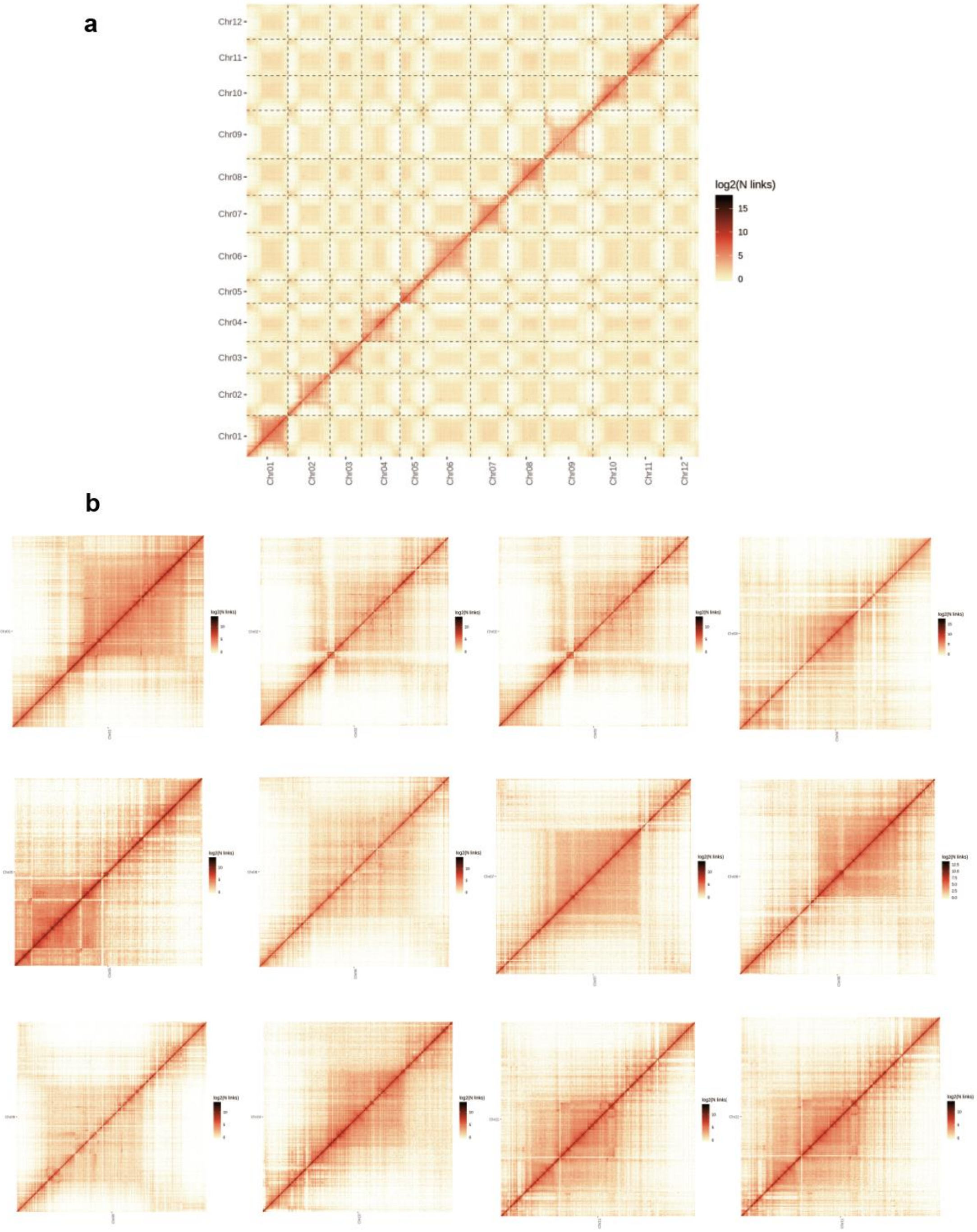

Supplementary Figure 3. Intensity heat map of Hi-C chromosome interaction for *A. elata* genome. a. Intergenomic intensity heatmap for *A. elata*; **b**. intensity signal heatmap for each chromosome. Darker red pixels denote higher contact probabilities. Most interactions were observed within the chromosomes.

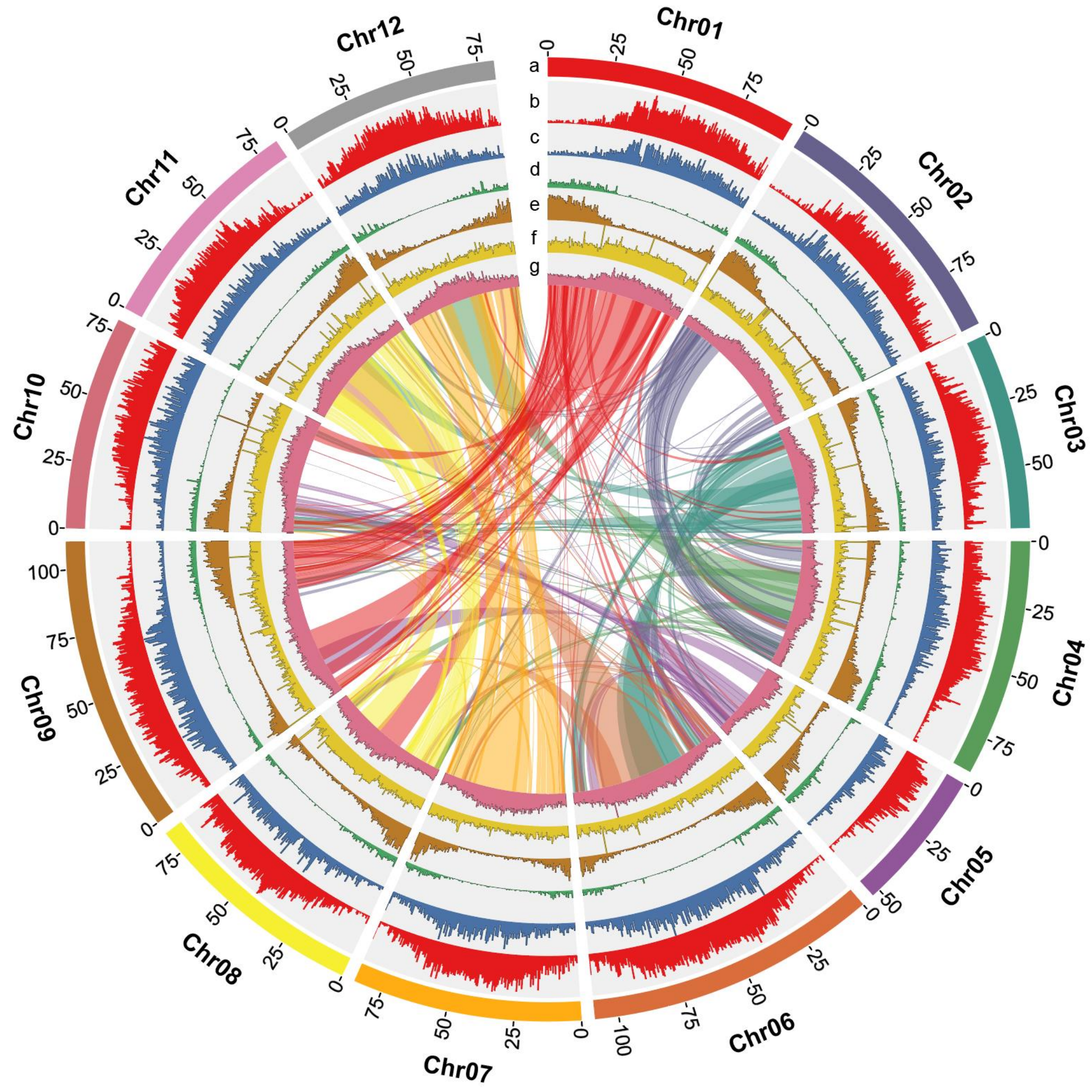

Supplementary Figure 4. Genome landscape of *A. elata*. a. pseudochromosomes number and length (on a Mb scale); **b**. distribution of Gypsy-type transposons (sliding window size 500 Kb); **c**. distribution of Copia-type transposons (sliding window size 500 Kb); **d**. distribution of DNA-type transposons (sliding window size 500 Kb); **e**. density of protein-coding genes (sliding window size 500 Kb); **f**. coverage of second-generation data; g. distribution of GC content (sliding window size 500 Kb). Each linking line in the center of the circle connects the colinear blocks. This Figure was generated using Circos (http://circos.ca/).

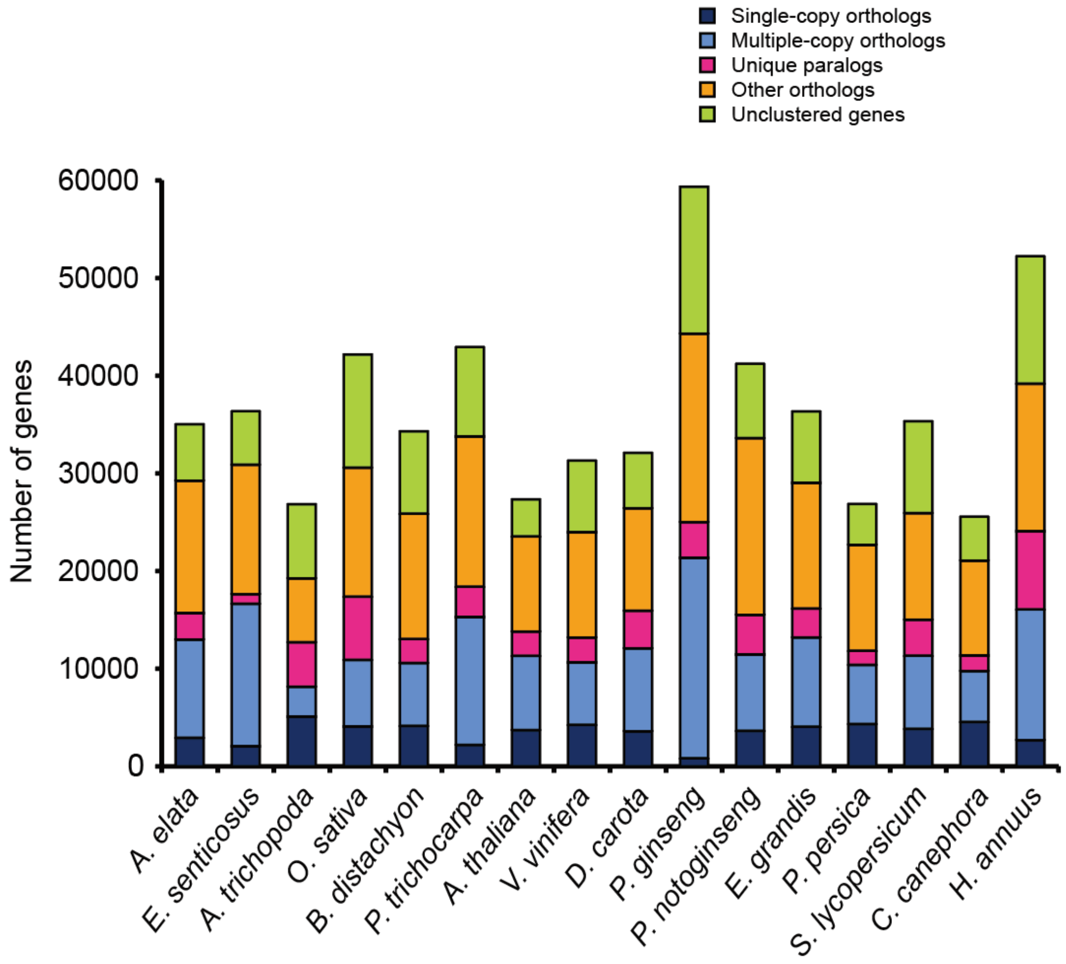

Supplementary Figure 5. Distribution of homologous genes in *A. elata* and other 15 plant species. Single-copy orthologs denotes genes with orthologs in all other species and no other paralogs in this species within one family. Multiple-copy orthologs refers to genes with orthologs in all other species and might have paralogs in the species of one family. Unique paralogs mean genes for which only one family contains genes of this species. Other orthologs describe genes not included in the other mentioned categories. Finally, unclustered genes are not clustered into any family.

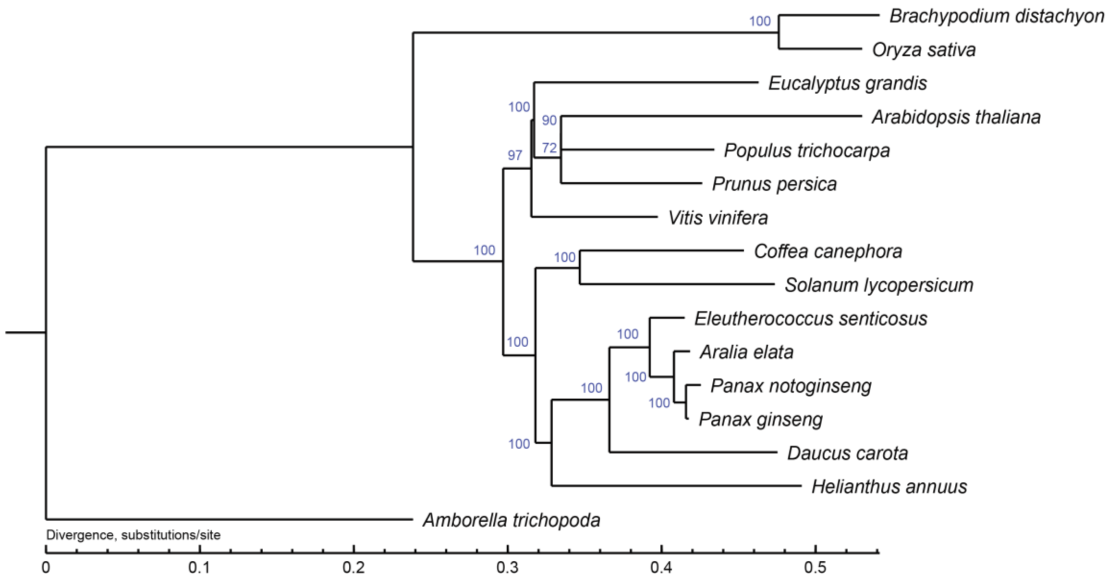

Supplementary Figure 6. The phylogenetic tree shows the topology and bootstrap values for *A. elata* and other 15 plant species. Branch length represents the rate of evolution. The numbers beside the branch indicated the bootstrap value (1000 times). MUSCLE was used to generate multiple sequence alignment for protein sequences in 145 single-copy families with default parameters. All the alignment results were combined to create a super alignment matrix. RAxML with GTRGAMMA model was used to construct phylogenetic tree with maximum-likelihood algorithms. *Amborella trichopoda* was designated as an outgroup of the phylogenetic tree.

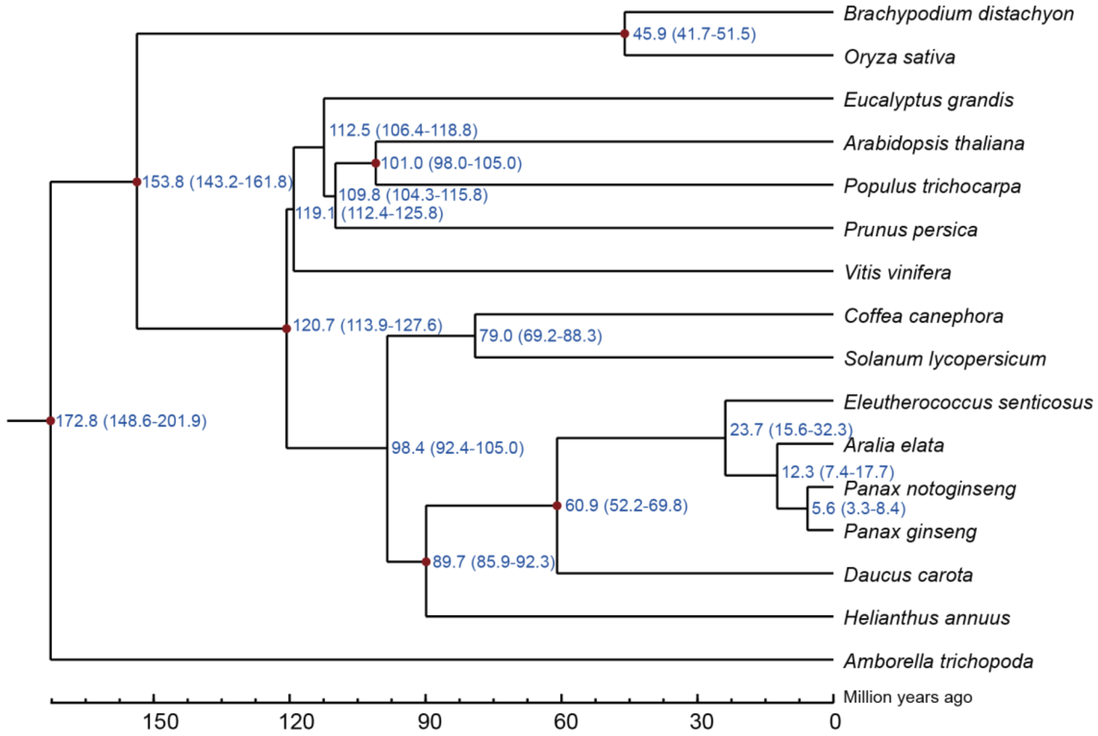

Supplementary Figure 7. Phylogenetic tree and divergence time estimation of *A. elata* and other 15 plant species. Divergence time was estimated using the mcmctree (http://abacus.gene.ucl.ac.uk/software/paml.html) embedded in the PAML package. The most likely divergence times from the most recent comment ancestor are given along each node, and the estimated ranges of divergence times are shown in the parentheses. Numbers in the parentheses are the predicted divergence times (95% confident intervals). The node dots indicate the calibration times of the divergence between *Brachypodium distachyon* and *Oryza sativa* (40.0-54.0 Mya), *Populus trichocarpa* and *Arabidopsis thaliana* (100.0-120.0 Mya), monocotyledons and dicotyledons (> 130.0 Mya), gymnosperms and angiosperms (< 200.0 Mya). The calibration times were retrieved from the TimeTree database (http://timetree.org).

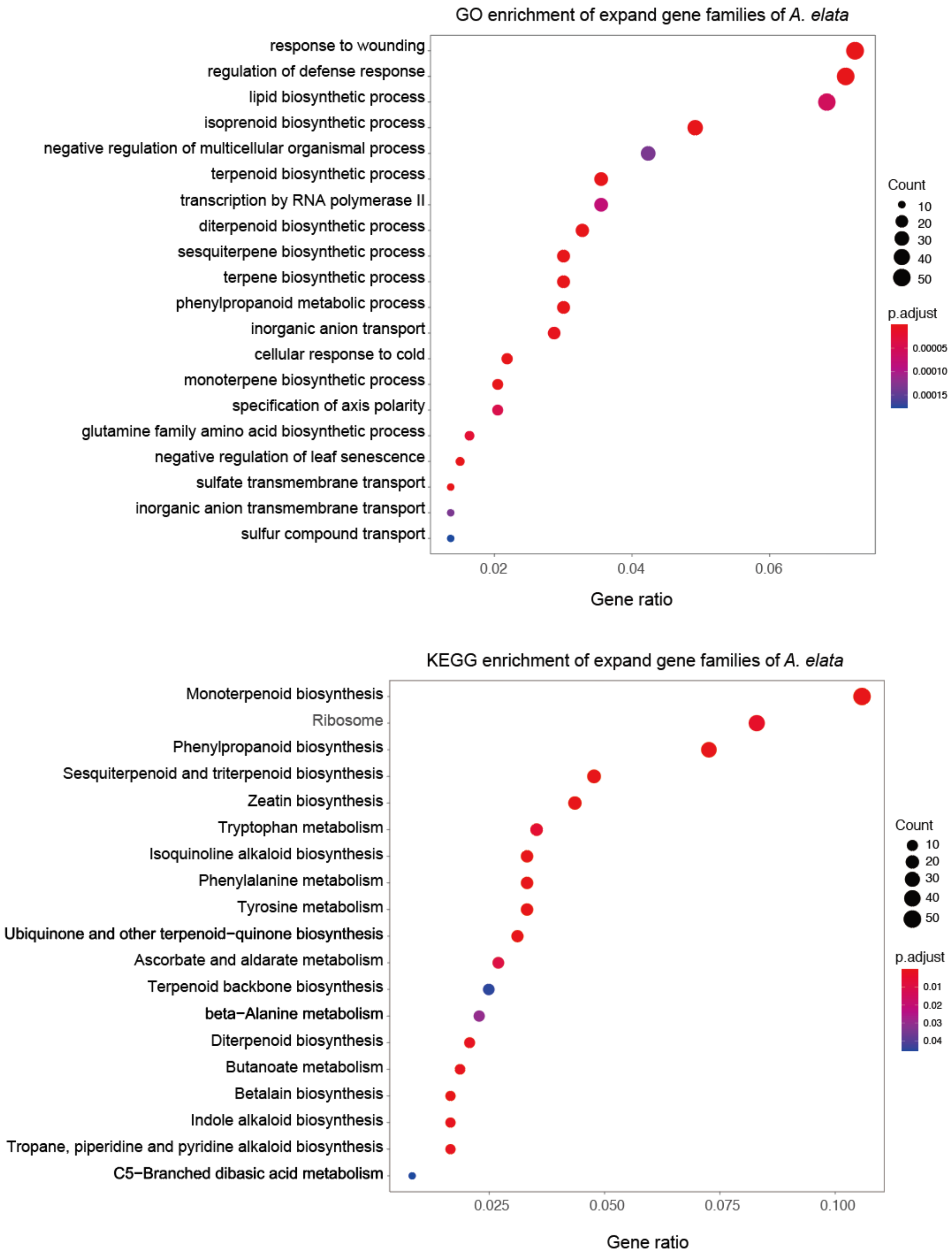

Supplementary Figure 8. GO and KEGG function enrichment of expanded gene families of *A. elata*. Circle sizes indicated gene numbers overlapped with the GO item group. Color indicates the adjust *P* values for hypergeometric test and FDR adjustments. Source data are provided as a Source Data file.

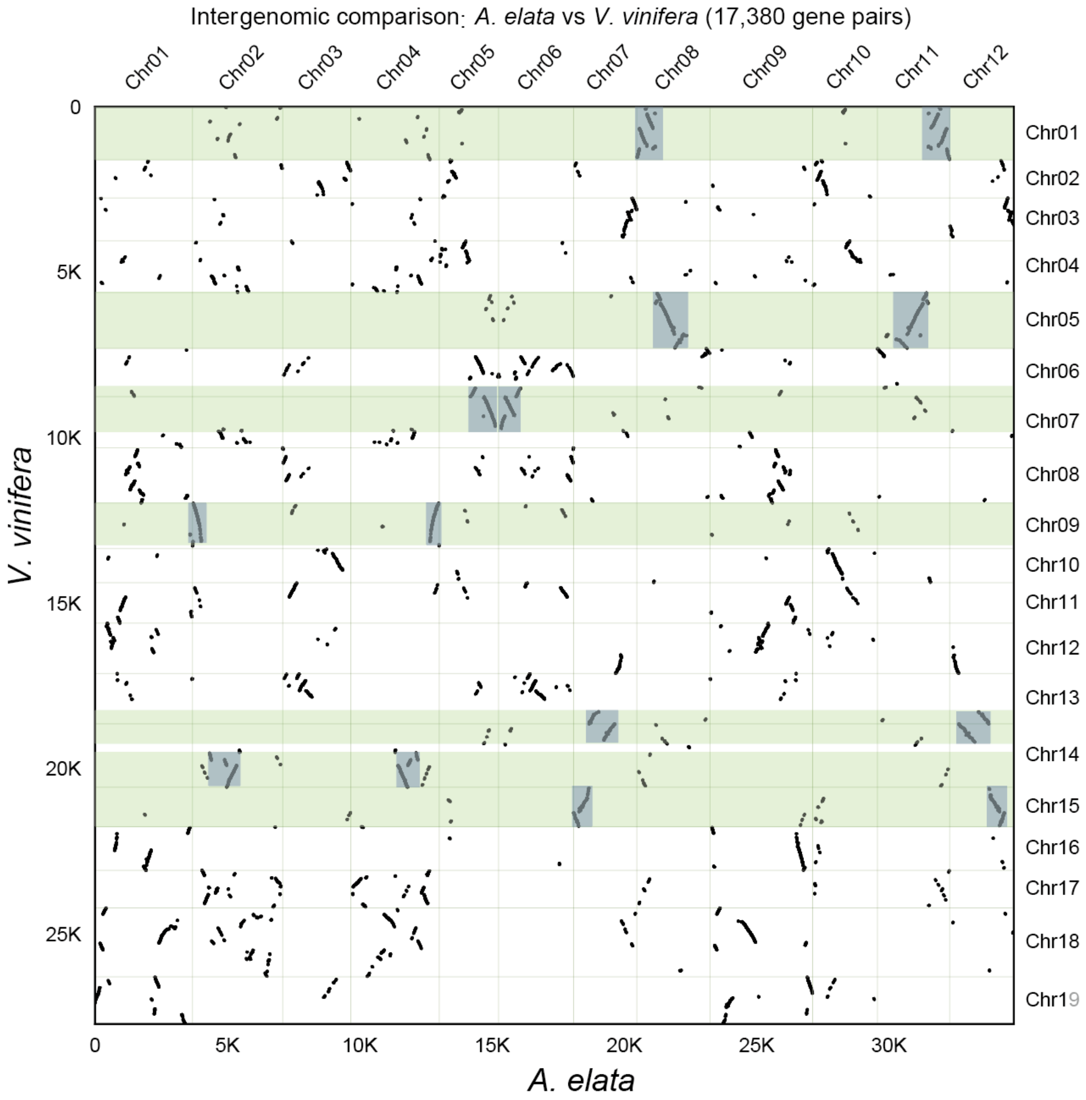

Supplementary Figure 9. Syntenic dot plots shows a 1:2 relationship between *A. elata* and *V. vinifera* genomes. The chromosome-level *V. vinifera* genome assembly (y axis) aligned to the chromosome-level *A. elata* genome assembly (x axis).

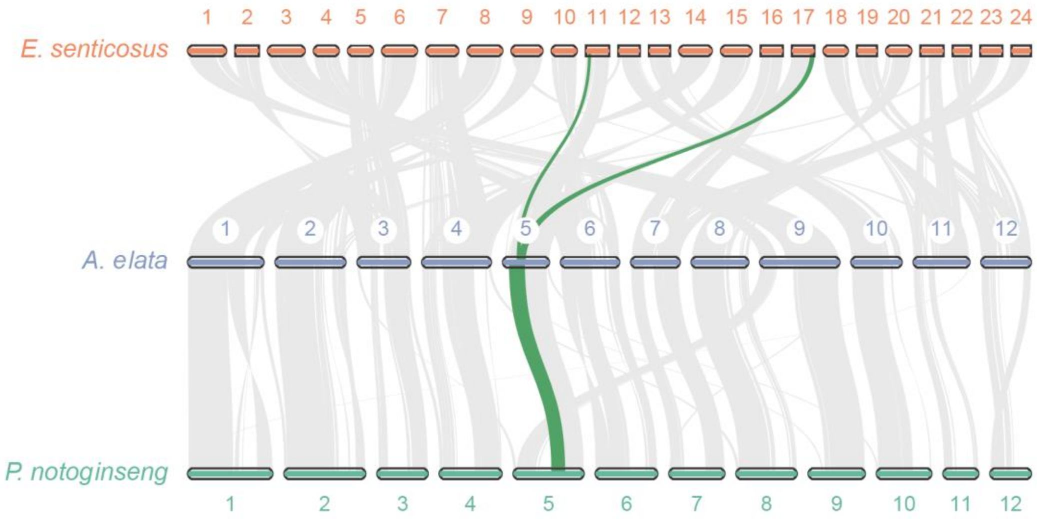

Supplementary Figure 10. Macrosynteny plot among *E. senticosus*, *A. elata* and *P. notoginseng* karyotypes. The marco-collinearity pattern shows that a typical ancestral region in the *P. notoginseng* genome can be traced to one region in *A. elata* and two regions in *E. senticosus*, respectively. Grey wedges in the background highlight major syntenic blocks spanning the genomes (highlighted by one syntenic set shown in green color).

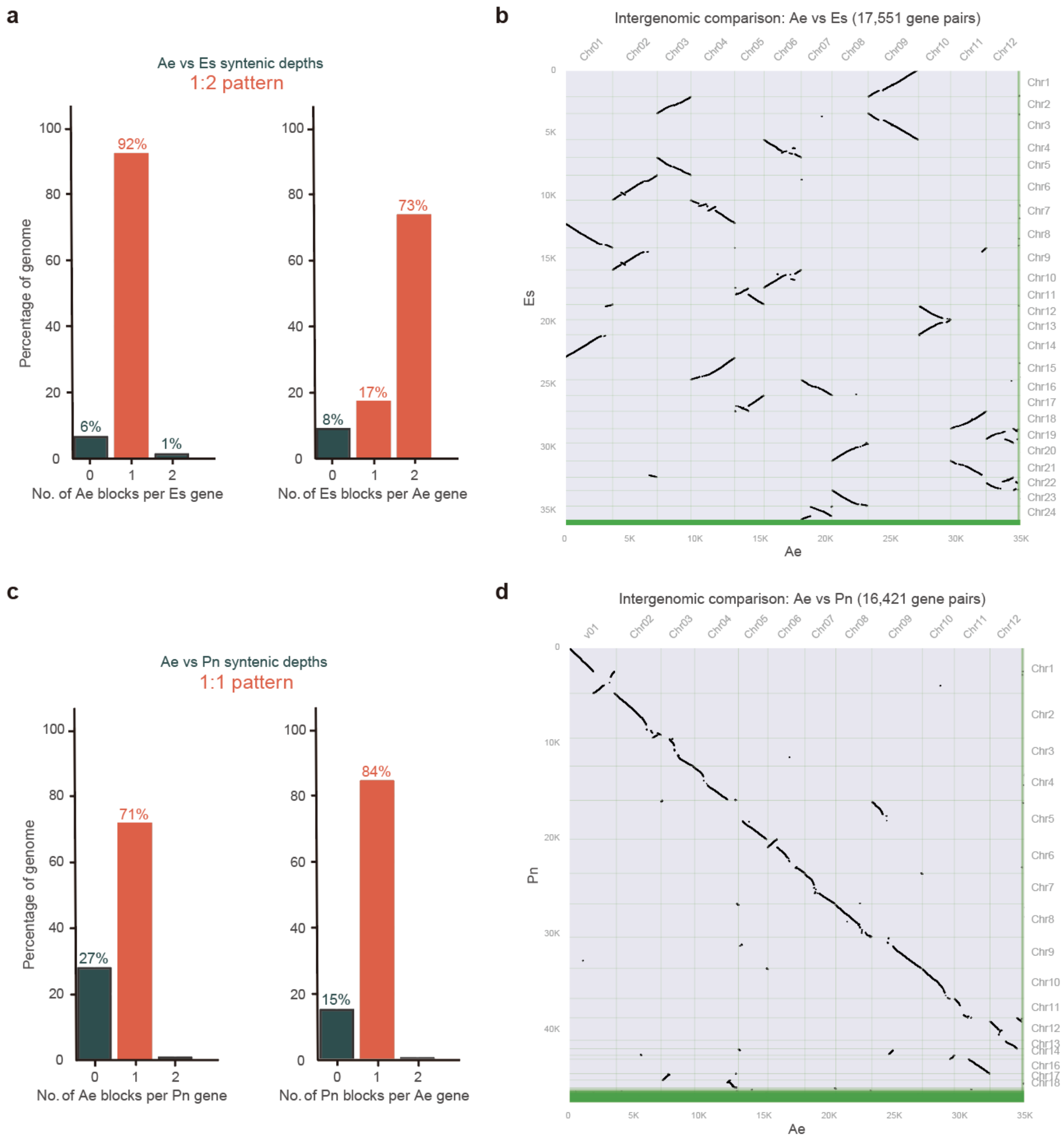

Supplementary Figure 11. Syntenic relationship of *A. elata*, *P. notoginseng* and *E. senticosus*. a. Syntenic depth showed 1:2 relationship between *A. elata* and *E. senticosus*. **b**. Intergenomic dot plot between *A. elata* and *E. senticosus*. c. Syntenic depth showed 1:1 relationship between *A. elata* and *P. notoginseng*. d. Intergenomic dot plot between *A. elata* and *P. notoginseng*. Ae, *A. elata*. Es, *E. senticosus*. Pn, *P. notoginseng*.

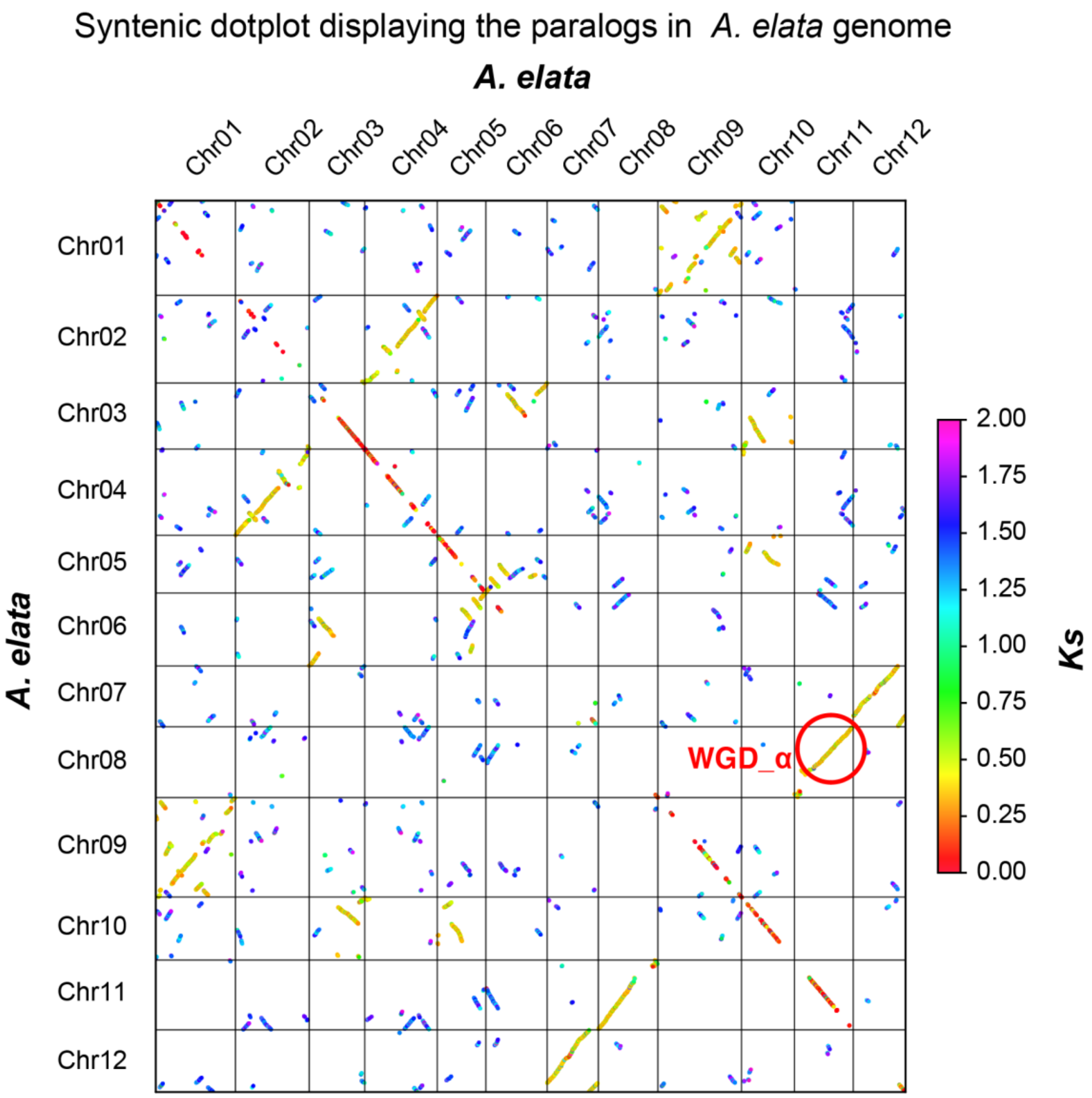

Supplementary Figure 12. Syntenic dotplot and *K*s dotplot displaying the paralogs in *A. elata* genome. Syntenic blocks were identified and colored based on their *K*s values. Representative paralogous regions were marked out by red circle.

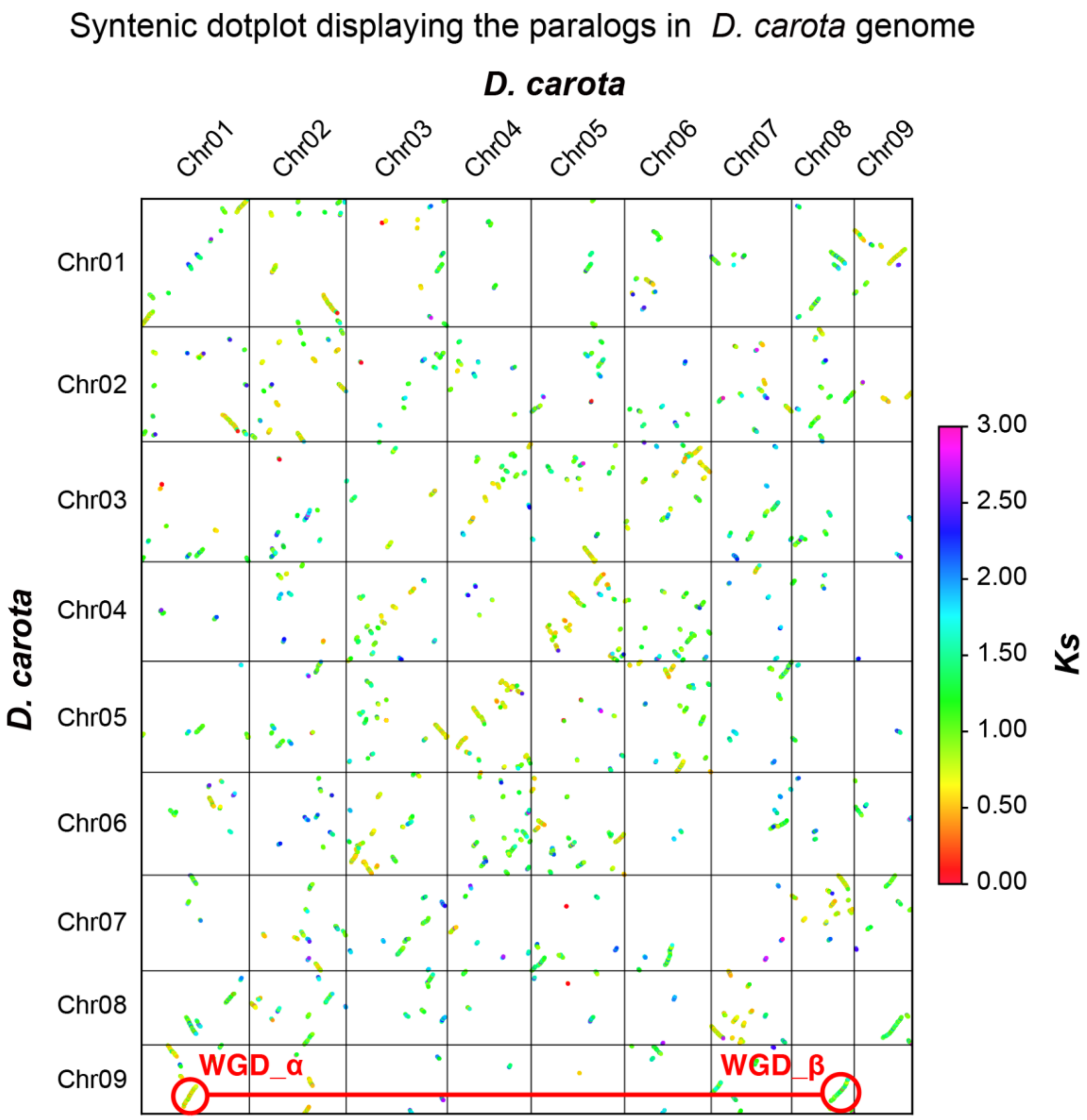

Supplementary Figure 13. Syntenic dotplot and *K*s dotplot displaying the paralogs in *D. carota* genome. Syntenic blocks were identified and colored based on their *K*s values. Representative paralogous regions were marked out by red circles.

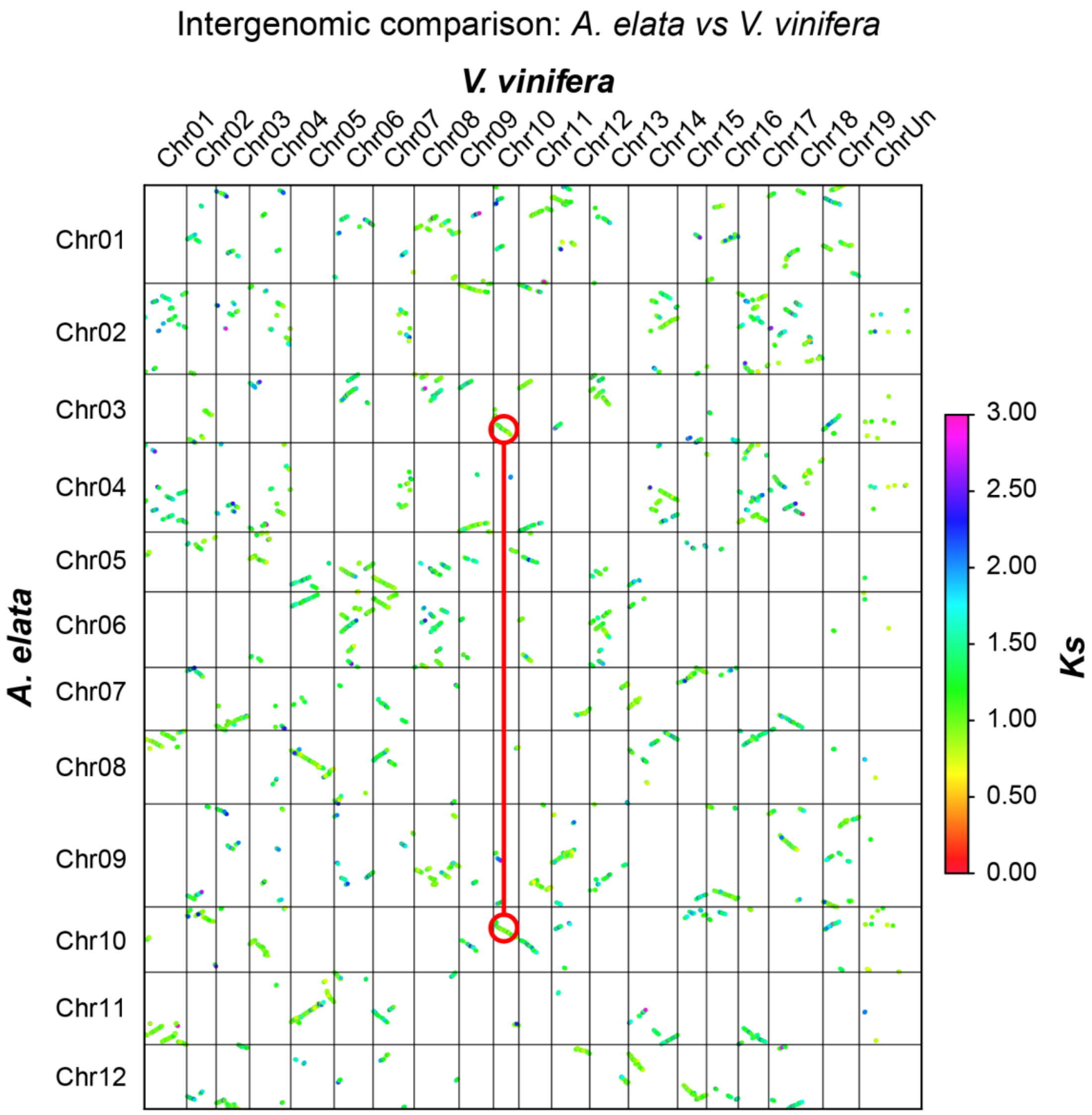

Supplementary Figure 14. Syntenic dotplot and *K*s dotplot displaying the orthologous between *A. elata* and *V. vinifera* genomes. Syntenic blocks were identified and colored based on their *K*s values. Representative orthologous regions were marked out by red circles.

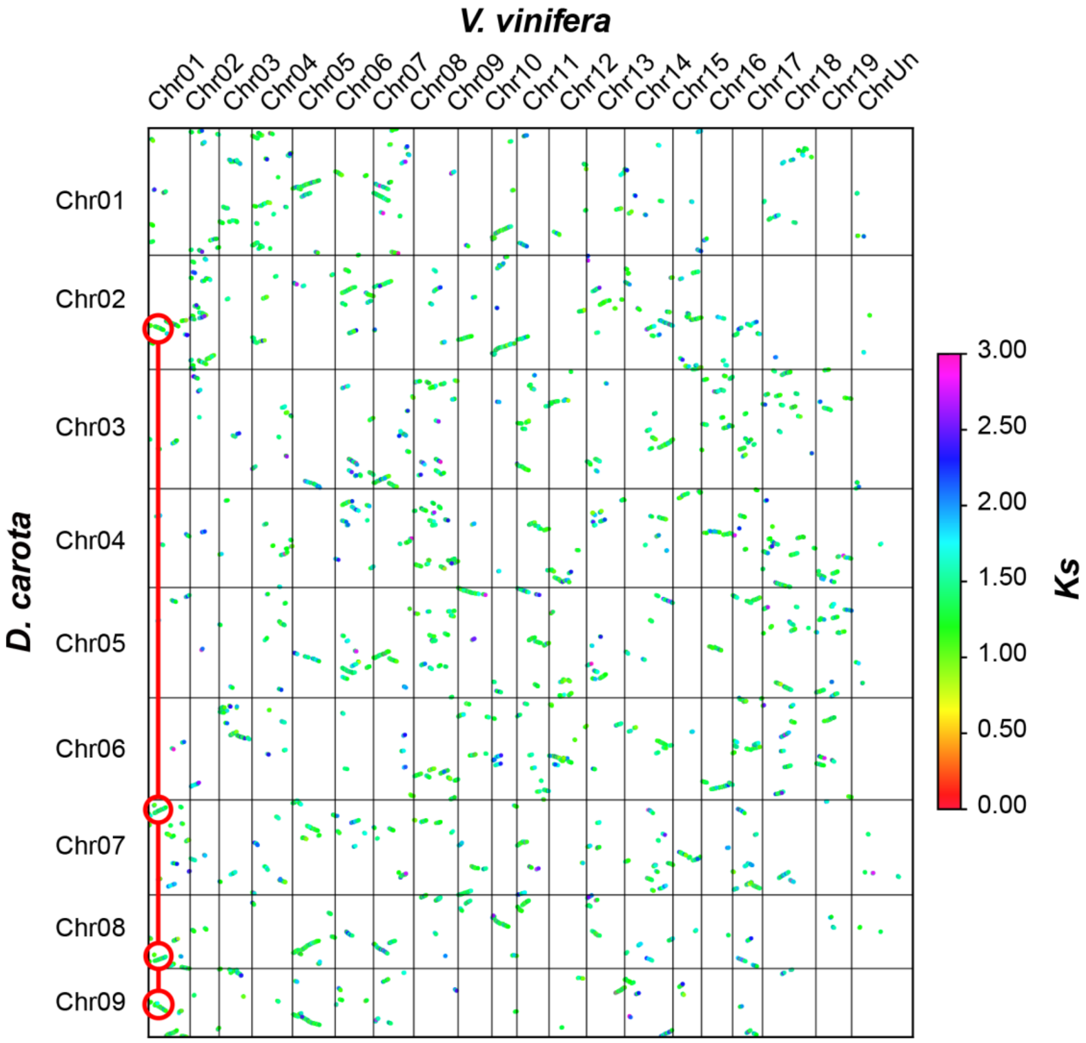

Supplementary Figure 15. Syntenic dotplot and *K*s dotplot displaying the orthologous between *D. carota* and *V. vinifera* genomes. Syntenic blocks were identified and colored based on their *K*s values. Representative orthologous regions were marked out by red circles.

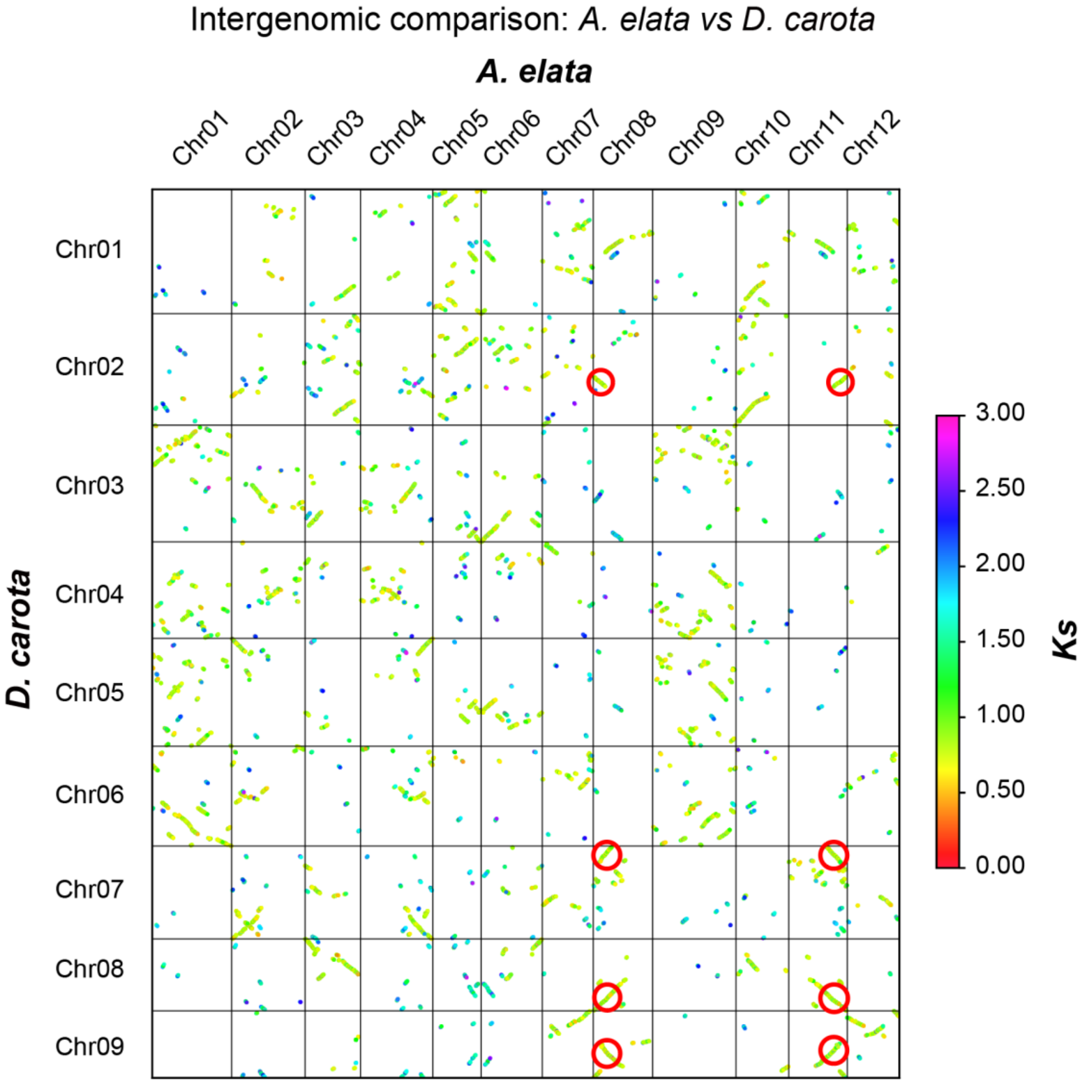

Supplementary Figure 16. Syntenic dotplot and *K*s dotplot displaying the orthologous between *A. elata* and *D. carota* genomes. Syntenic blocks were identified and colored based on their *K*s values. Representative orthologous regions were marked out by red circles.

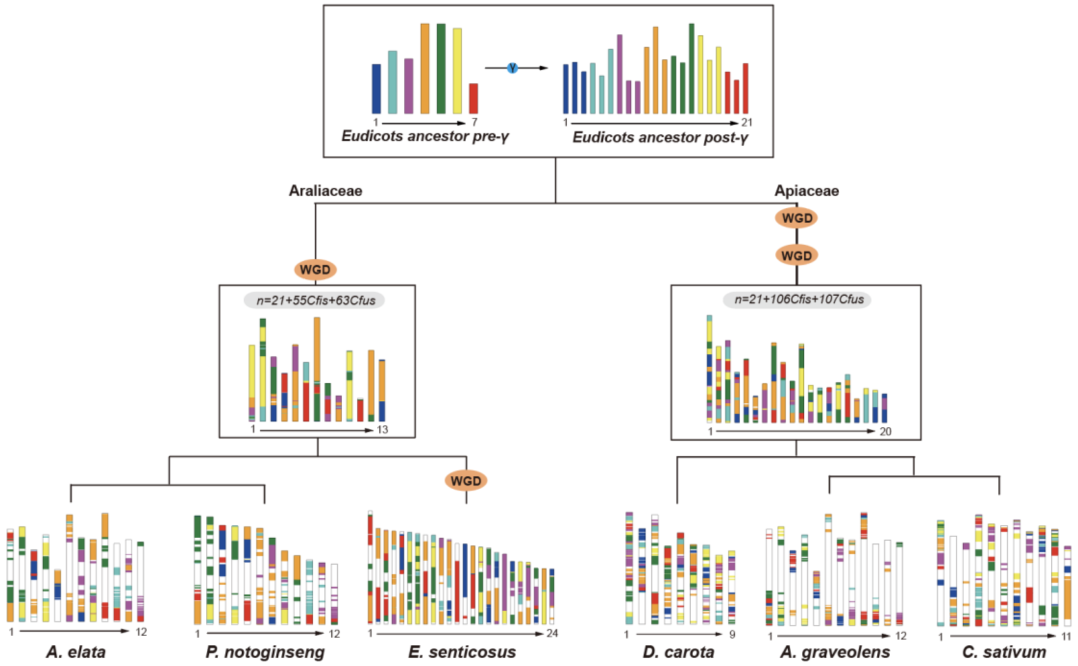

Supplementary Figure 17. Evolutionary scenario of the Apiales (*A. elata, P. notoginseng, E. senticosus, D. carota, A. graveolens,* and *C. sativum*) from the reconstructed ancestral eudicots karyotypes (AEKs) of 21 (post-WGT-γ) and 7 (pre-WGT-γ) proto-chromosomes. The modern genomes are illustrated at the bottom with different colors reflecting the origin from the seven ancestral chromosomes from AEK. Polyploidization events are shown with orange and blue solid ellipses. Cfis indicated the fission of chromosomes. Cfus indicated the fusion of chromosomes.

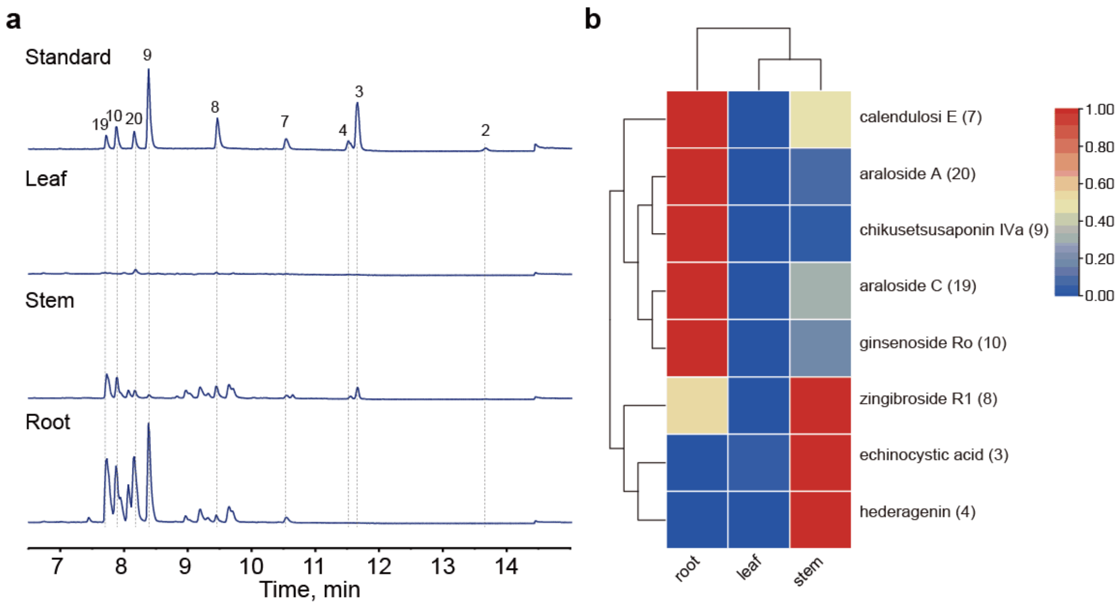

Supplementary Figure 18. Contents of pentacyclic triterpenes in three organs of *A. elata*. a. Overlays of LC–MS chromatograms for roots, stems and leaves of 5-year-old *A. elata* obtained by extract ions chromatogram (EIC) of the theoretical *m*/*z* values of the compounds of interest. The number of standards representing echinocystic acid (3), hederagenin (4), calenduloside E (7), zingibroside R1 (8), chikusetsusaponin IVa (9), ginsenoside Ro (10), araloside C (19), and araloside A (20) are shown, respectively. **b**. Peak area of each compound obtained by EIC were normalized, and the heatmaps generated based on hierarchical clustering of the accumulation profiles. Source data underlying Supplementary Figure 18b are provided as a Source Data file.

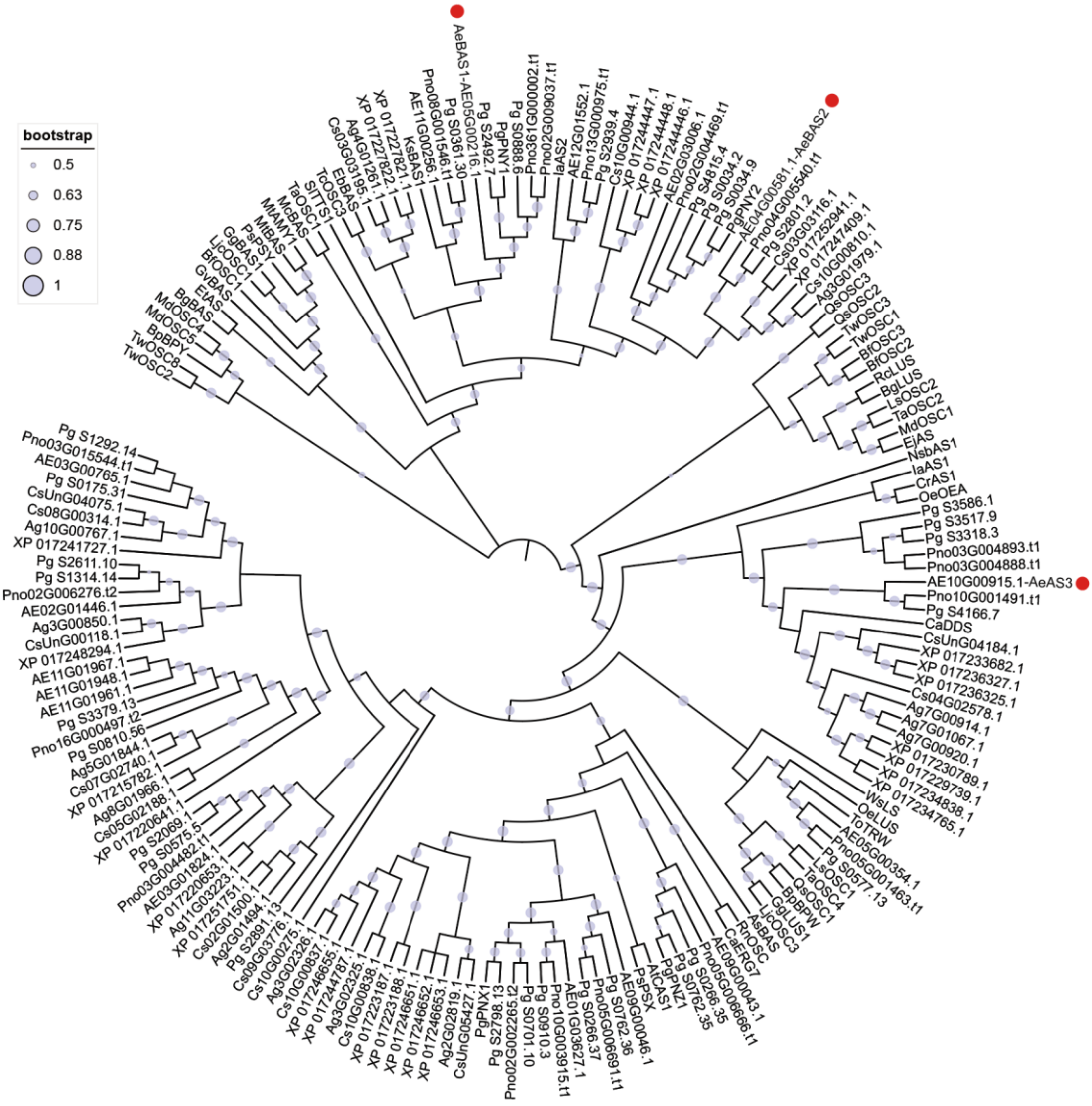

Supplementary Figure 19. Phylogenetic tree of genes from OSC family in different species using the maximum likelihood method (1,000 bootstraps). Red dots indicate the genes analyzed in this study.

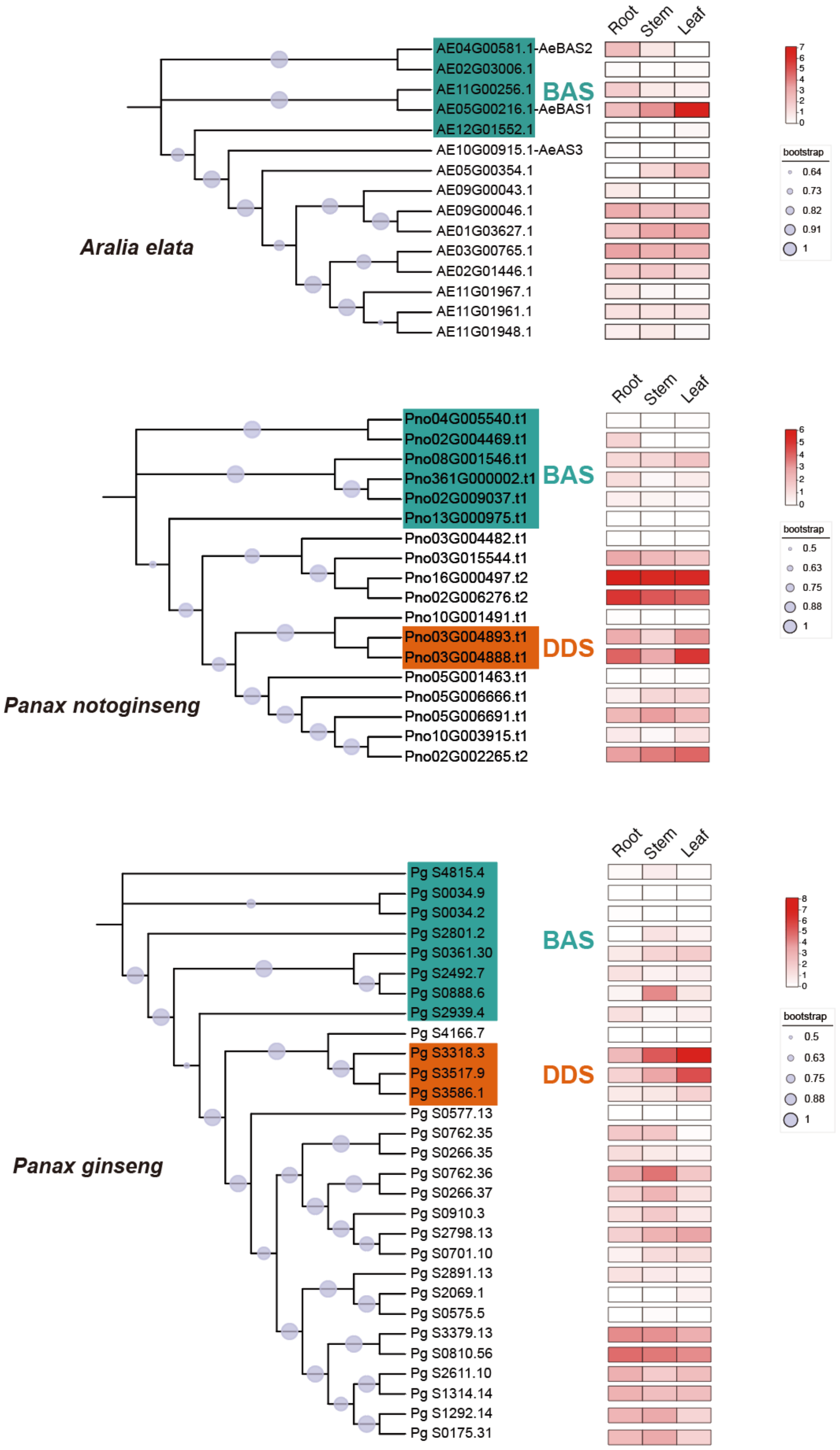

Supplementary Figure 20. Phylogenetic tree and expression profile (FPKM) for genes in OSC gene family in *P. ginseng*, *P. notoginseng*, and *A. elata*. The tree was constructed using the maximum likelihood method (1,000 bootstraps, light purple circles). Source data are provided as a Source Data file.

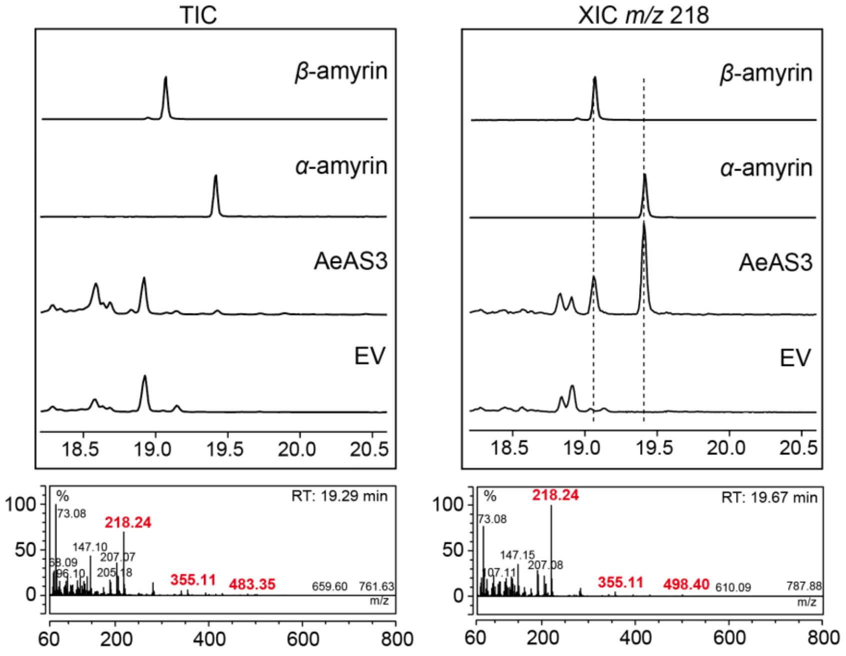

Supplementary Figure 21. *In vivo* enzymatic function identification of AeAS3. GC–MS chromatograms obtained by total ions chromatogram (TIC) and extract ions chromatogram (EIC) of the metabolites from yeast strains expressing the *AeAS3* or containing empty vector. The characteristic peak of the β-amyrin and α-amyrin (*m*/*z* 218) was extracted in EIC. The MS/MS chromatograms of new products were shown in the box below, and the characteristic peaks of corresponding compound were marked by red color. The strain containing the empty vector (EV) was as the negative control.

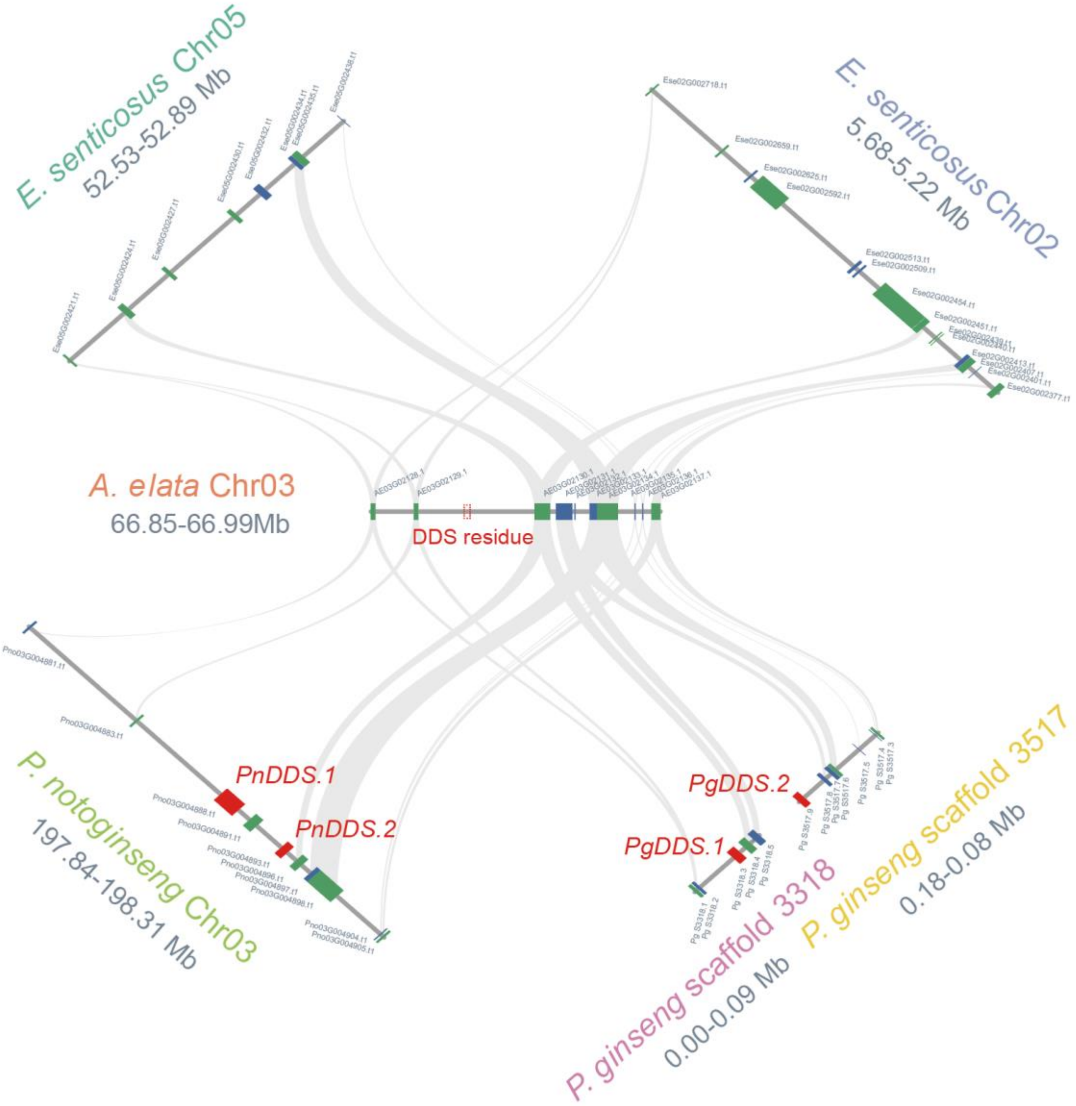

Supplementary Figure 22. Synteny analysis of *DDS* genes between *A. elata, E. senticosus, P. notoginseng,* and *P. ginseng*. The red box indicated *DDS* genes in *P. notoginseng* and *P. ginseng*. The green and blue box indicated forward and reverse directions of genes on chromosomes, respectively. Grey wedges in the background highlight syntenic genes spanning the genomes.

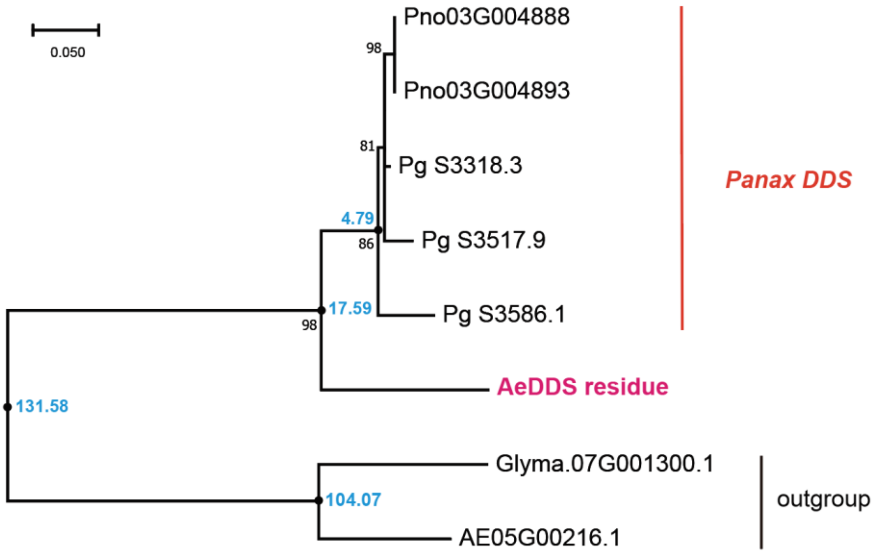

Supplementary Figure 23. *Ks* and phylogenetic analysis of *AeDDS* residues with *DDS* from *P. notoginseng* and *P. ginseng*. The blue numbers beside the node indicate the divergence time (million years ago) of every clade. The divergence time were calculated by T=*K*s/2r, where r represents the common substitution rate 6.5×10^{-9} for eudicots. The tree was constructed using the maximum likelihood method (1,000 bootstraps). Pg, *P. ginseng*. Pno, *P. notoginseng*. AE, *A. elata*. Glyma, *Glycine max*. Source data are provided as a Source Data file.

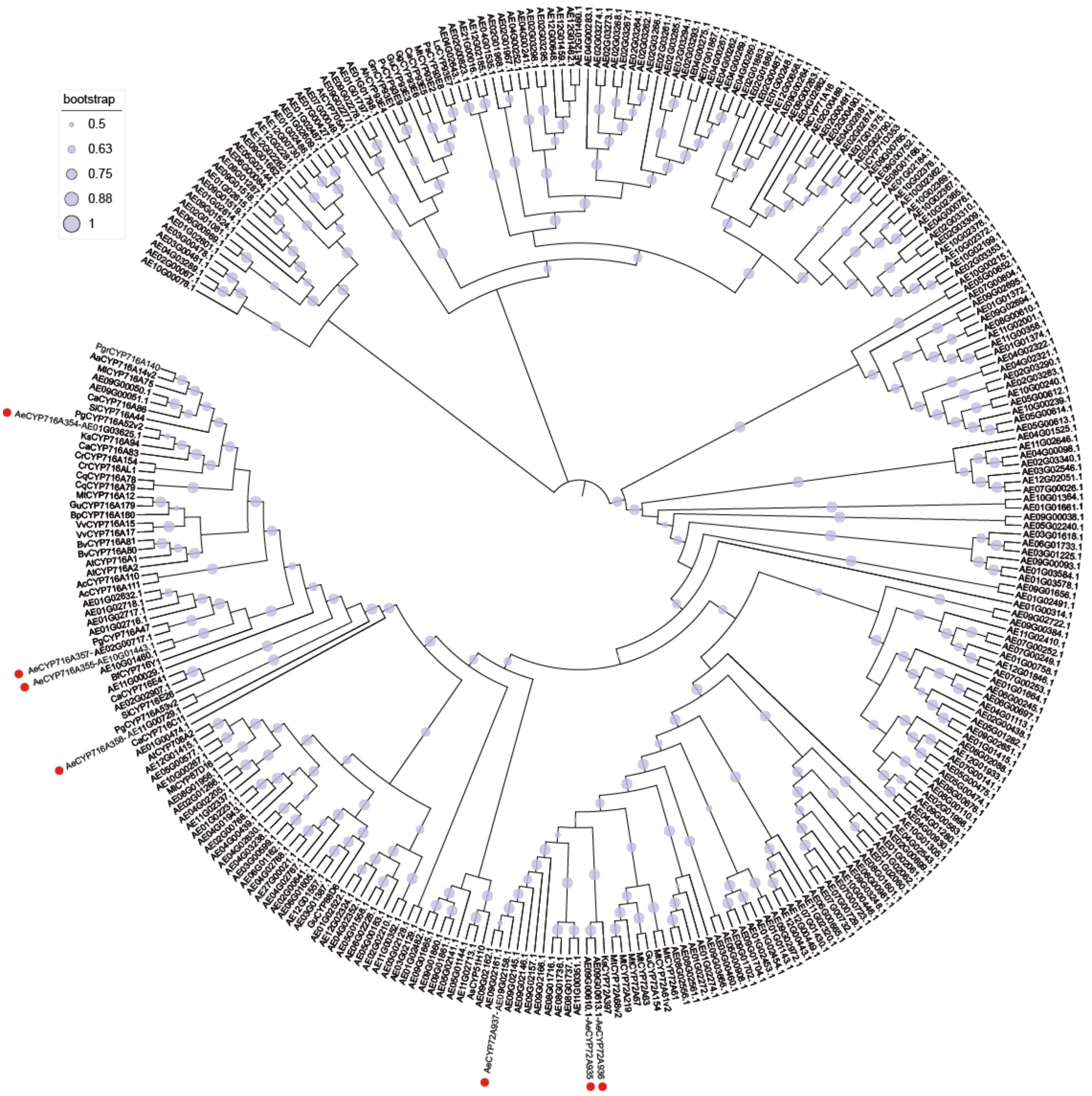

Supplementary Figure 24. Phylogenetic tree of genes from P450 family in different species. Red dots indicate the tandem repeat genes involved in this study. The tree was constructed using the maximum likelihood method (1,000 bootstraps).

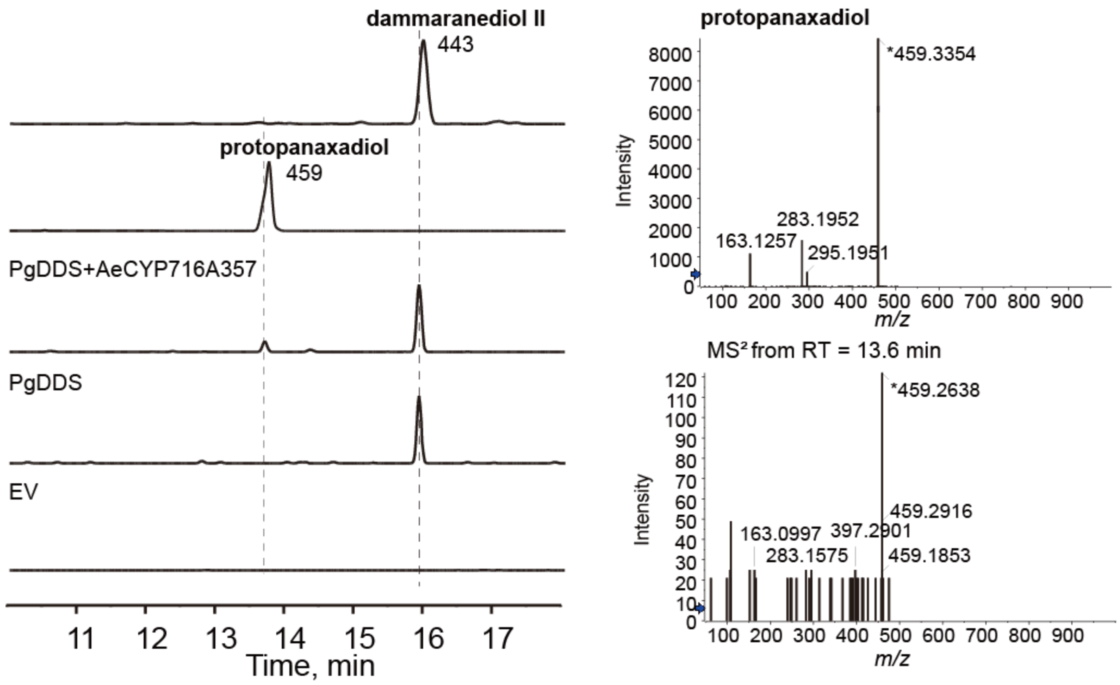

Supplementary Figure 25. LC-MS indicates that AeCYP716A357 is the functional protopanaxadiol synthase in yeast. Overlays of LC–MS chromatograms obtained by extract ions chromatogram (EIC) of the theoretical *m*/*z* values of the compounds of interest. Extract ion chromatogram overlay of the yeast strains expressing the *PgDDS* and *AeCYP716A357*, or without the *AeCYP716A357* as control. Peaks potentially corresponding to saponins are labeled with the *m*/*z* value of protopanaxadiol (*m*/*z* 459), and the MS/MS spectra are shown in the right panel.

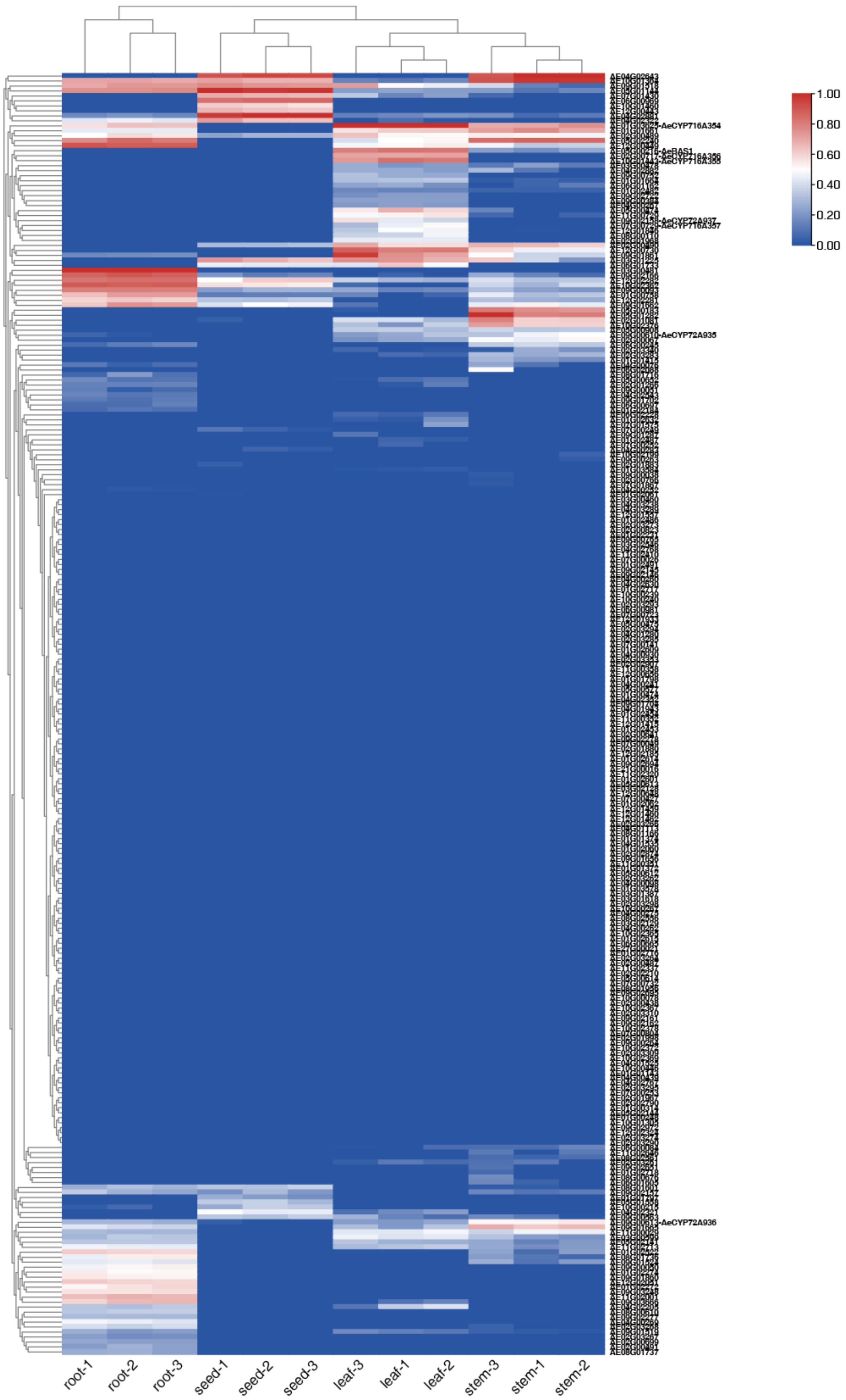

Supplementary Figure 26. Expression pattern (FPKM) of 256 *P450* genes in leaves, stems, roots, and seeds of *A. elata*. The heatmaps show hierarchical clustering of expression profiles with normalized expression levels. Source data are provided as a Source Data file.

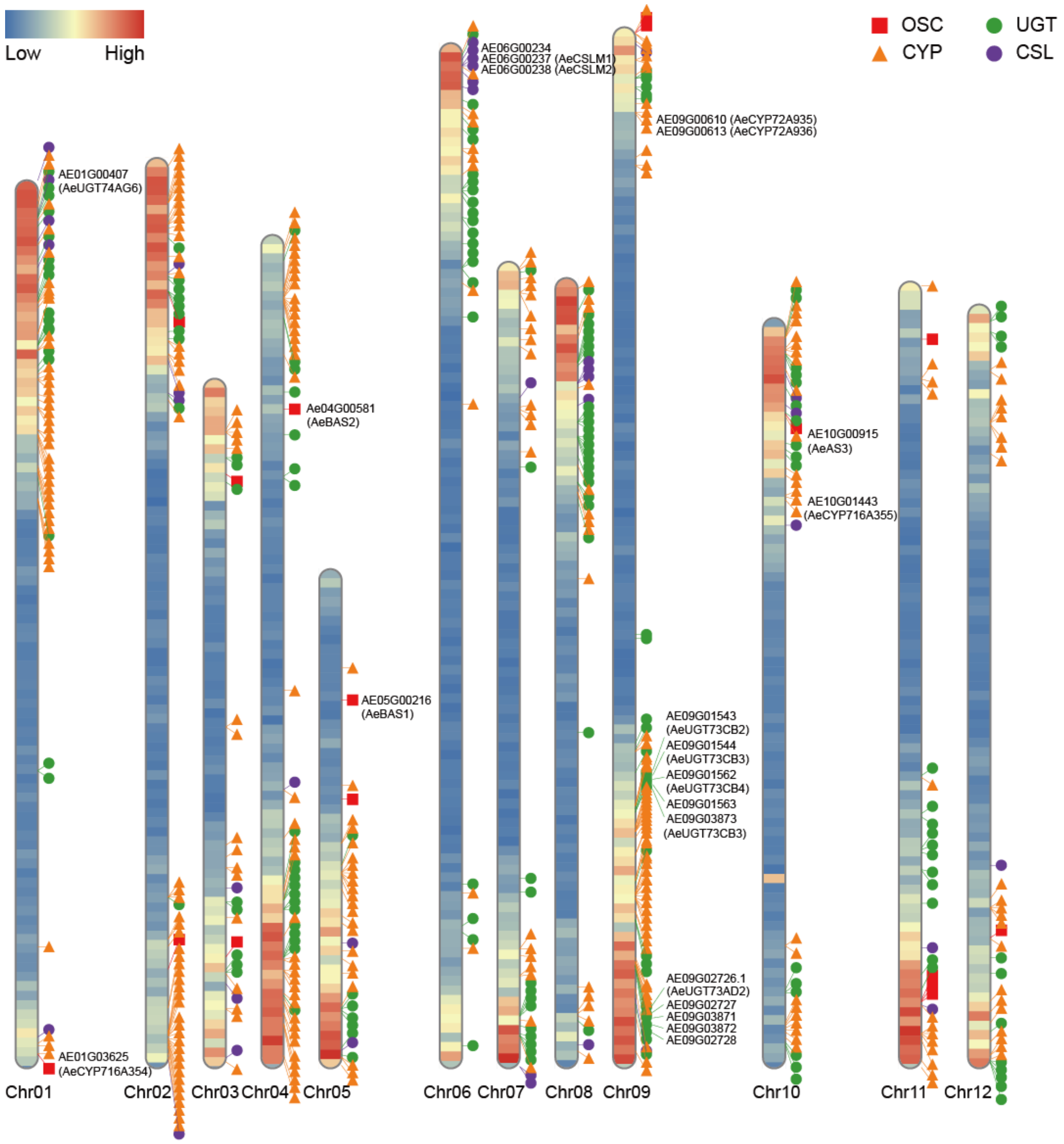

Supplementary Figure 27. Distribution of *OSC, P450, UDPGT,* and *CSL* genes on *A. elata* chromosomes. The color on the chromosomes indicates gene density. The sliding window size is 1 Mb.

```
AeCYP72A936-AE09G00613.1    MEMD-IVFYLSIAVIVGIVAWAWRVLNWVWLRPKKVEKLVRKQGFHGNSYRLLSGDMKQS 59
AeCYP72A935-AE09G00610.1    MEMDRVVFYLSIAVIVGIVAWAWRVLNWVWLRPKKVEKLLRKQGFHGNSYRLLYGDMKQD 60
Pno09G000253.t1             --MA-AAAAVAVAAVV-FVGWAWKVLDWVWVSPRKLEKSLRKQGFRGNSYRLFHGDQKEH 56
Clustal Consensus             *   .  :::*.:* :*.***:**:***: *:*:** :*****:******: ** *:

AeCYP72A936-AE09G00613.1    AWLIRDAKSKPIHVSDNIAPRVIPLVSHIINNYGKKSFVWVGPTLRVHIMDPELIKEVFS 119
AeCYP72A935-AE09G00610.1    AWMIKDAKSKPIHVSDNIAPRVIPLVSHIINNYGKKSFVWFGPALRVIIMDPELIKEVFT 120
Pno09G000253.t1             SEMTRKATLKPINLSDEVVLRLRPFIHQTLHKYGKKSFIWIGPTPRVHIMDPELIKEILV 116
Clustal Consensus           : : :.*. ***::**::. *: *:: : :::******:*.**: ** *********::  90

AeCYP72A936-AE09G00613.1    KNNKFQKPVLNPLFKLFTNGLPTIEGDKWDKHRKLLNPAFHLEKLKCMVPTFYLCCCEMT 179
AeCYP72A935-AE09G00610.1    KHNKFQKPILNPLFKLFSNGLSSIEGEKWAKHRKILNPAFHLEKLKCMVPTFYLCCCEMT 180
Pno09G000253.t1             KSSKFNKPKRNPLIKLFADGLANHDAELWAKHRKLLNPAFHIEKLKCMLPAMYLSCIEMV 176
Clustal Consensus           * .**:**  ***:***::**.. :.: * ****:******:******:*::**.* **. 137

AeCYP72A936-AE09G00613.1    RKWEEMMLEGASCELDVWPFLQTFARDVISRTGFGSSYKEGSRVFELQIEQAQLAIKSMQ 239
AeCYP72A935-AE09G00610.1    RKWEEMMLEGASCELDVWPFLQTFSRDVISRNAFGSSYKEGSRIFELLIEQTKLAIKSVQ 240
Pno09G000253.t1             SKWEKMISEDGSCELDVWPFLHRLTKDVISHTAFGSSYEEGNIVFELQTEQAELVMKTLL 236
Clustal Consensus            ***:*: *..**********: :::****:..*****:**. :***  **::*.:*::

AeCYP72A936-AE09G00613.1    SVYIPGVRYLPTKRNQRMKAIEREIHFSLKSIINKKMEAIKAGKGSSDDLLGILLESNFN 299
AeCYP72A935-AE09G00610.1    SVYIPGARYLPTKRNQRMKAIEREMQFSLKSIINKKMEAINAGKGANDDLLGILLESNFN 300
Pno09G000253.t1             SVYIPGWSYLPTKRNKRMKEIDRKTQSCLMNIINKKTKAMQAGEATADDLLGILLESNLK 296
Clustal Consensus           ******  *******:*** *:*: : .* .***** :*::**:.: ***********:: 233

AeCYP72A936-AE09G00613.1    --VQHGNKNVGMSAQDVIEECKVFYFAGQETTSVLLVWTMVLLSIHPNWQAQARKEVIQV 357
AeCYP72A935-AE09G00610.1    E-VRHGNKNVGMSAQDVIEECKLFYFAGQESTSVLLVWTMVLLSIHPNWQAQARKEVLQV 359
Pno09G000253.t1             EQLGQGNKNVGMSIQEVMGECKQFYFAGQETTSGLLVWTMVLLSIHPDWQKRAREEILQE 356
Clustal Consensus             : :******** *:*: *** *******:** *************:** :**:*::*

AeCYP72A936-AE09G00613.1    IGNGKLDFEDLSQLKIVTMILYEVLRLYPPVALLVRRVNEETTLGDITLPPGVEVSLPII 417
AeCYP72A935-AE09G00610.1    IGNGKLDFEDLSQLKIVTMILYEVLRLYPPVPLLARRVHEETTLGDITLPPGVEVSLPII 419
Pno09G000253.t1             FGNEKPDFDHLNHLKIVTMILYEVLRLYPPIDTLFRRVDQETTLGDITLP-GVQISIPLM 415
Clustal Consensus           :** * **:.*.:*****************:  * ***.:********** **::*:*:: 335

AeCYP72A936-AE09G00613.1    LHHHDPQIWGDDAKEFKPERFSEGVSKATQNQVMFFPFGGGPRICIGQNFAMLEAKLAIA 477
AeCYP72A935-AE09G00610.1    LYHHDPQIWGDDVKEFNPERFSQGVLKATQNQVMFFPFGGGPRICIGQNFAMLEAKLAIA 479
Pno09G000253.t1             VLHRDQNIWGDDAREFNPERFSEGVSKATKNQVVFFPFGWGPRICIGQNFAMLEAKLALA 475
Clustal Consensus           : *:* :*****.:**:*****:** ***:***:***** ******************:* 390

AeCYP72A936-AE09G00613.1    TILTKFSFELSPSYTHAPLNLFTVQPQYGANLILHKI 514
AeCYP72A935-AE09G00610.1    TILTKLSFELSPSYTHAPLNLFTVQPQYGANLILHKI 516
Pno09G000253.t1             VILQRFSFELSPSYTHAPINVLTVQPQHGANLILHKL 512
Clustal Consensus           .** ::************:*::*****:********: 425
```

Supplementary Figure 28. Alignment of amino acid sequence of two AeCYP72A members and the collinear P450 enzyme in *P. notoginseng*.

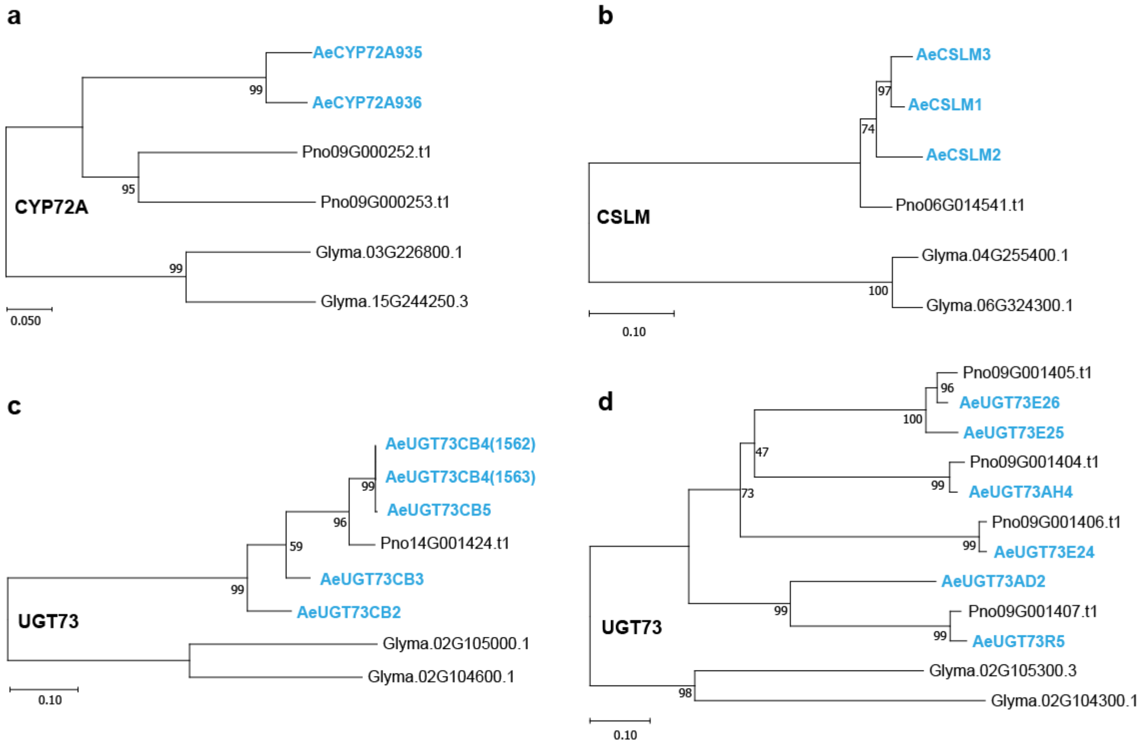

Supplementary Figure 29. The phylogenetic tree of genes in Figure 5. a. The phylogenetic tree of genes in Figure 5a. **b**. The phylogenetic tree of genes in Figure 5b. **c**. The phylogenetic tree of genes in Figure 5c. **d**. The phylogenetic tree of genes in Figure 5d. The blue letters indicate the tandem repeat genes in *A. elata* genome. The numbers near the nodes represent the bootstrap value. The numbers under/over the branches represent the branch lengths. AE, *A. elata*. Pno, *P. notoginseng*. Glyma, *Glycine max*. Source data are provided as a Source Data file.

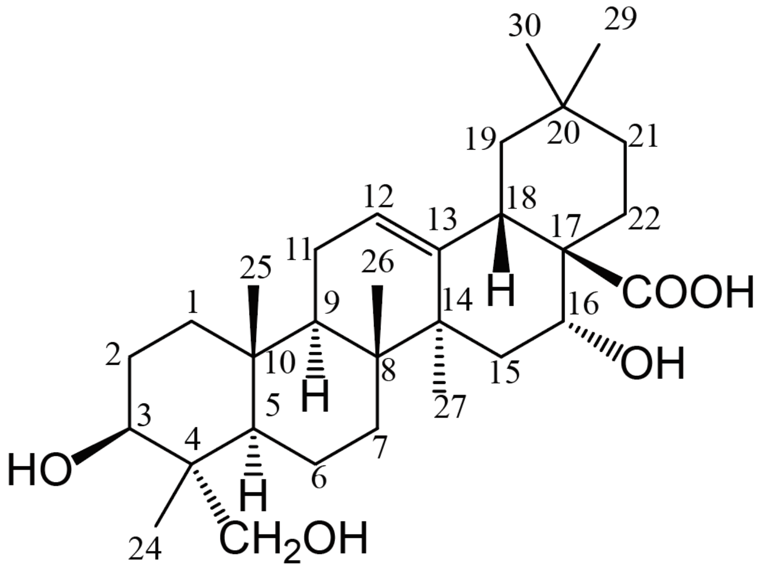

Supplementary Figure 30. Structure of 16-OH-hederagenin identified by NMR.

16-OH-hederagenin, MS $(M\text{-}H)^-$ *m/z* 487.34, calcd for $C_{30}H_{48}O_5$ 488.35. ^1H NMR (800 MHz) δ : 3.07 (dd, J_1 = 6.6 Hz, J_2 = 3.3 Hz, 1 H, H-3), 4.14 (s, 1 H, H-3, OH), 5.20 (t, J_1 =2.8 Hz, 1 H, H-12), 4.70 (d, J_1 =2.8 Hz, 1 H, H-16), 2.88 (dd, J_1 =9.0 Hz, J_2 =2.8 Hz, 1 H, H-18), 1.91 (m, 2 H, H-19), 3.43 (m, 1 H, H-23), 4.32 (d, J_1 =2.3 Hz, 1 H, H-23), 4.38 (s, 1 H, H-23, OH), 11.99 (s, 1 H, H-23, -COOH); ^1C NMR (800 MHz) δ : C-1 (J_1 =37.96 Hz), C-2 (J_1 =26.49 Hz), C-3 (J_1 =70.24 Hz), C-4 (J_1 =41.84 Hz), C-5 (J_1 =47.26 Hz), C-6 (J_1 =17.49 Hz), C-7 (J_1 =32.21 Hz), C-8 (J_1 =38.86 Hz), C-9 (J_1 =46.47 Hz), C-10 (J_1 =36.28 Hz), C-11 (J_1 =22,85 Hz), C-12 (J_1 =121.26 Hz), C-13 (J_1 =144.00 Hz), C-14 (J_1 =42.15 Hz), C-15 (J_1 =35.25 Hz), C-16 (J_1 =72.93 Hz), C-17 (J_1 =46.17 Hz), C-18 (J_1 =40.80 Hz), C-19 (J_1 =46.39 Hz), C-20 (J_1 =30.23 Hz), C-21 (J_1 =34.65 Hz), C-22 (J_1 =31.45 Hz), C-23 (J_1 =64.40 Hz), C-24 (J_1 =12.66 Hz), C-25 (J_1 =15.57 Hz), C-26 (J_1 =16.88 Hz), C-27 (J_1 =26.57 Hz), C-28 (J_1 =178.17 Hz), C-29 (J_1 =32.87 Hz), C-30 (J_1 =24.15 Hz).

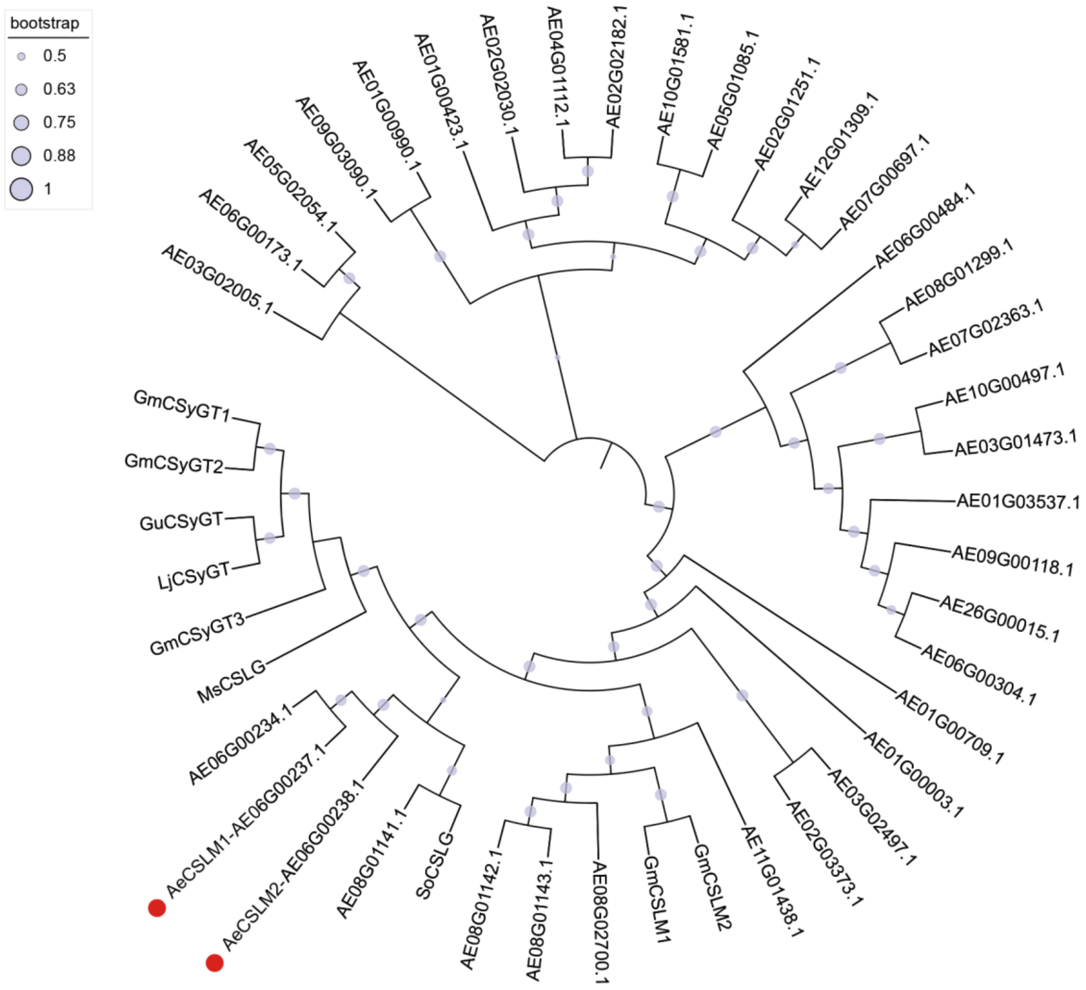

Supplementary Figure 31. Phylogenetic tree of CSL gene family in different species. Red dots indicate tandem repeat genes analyzed in this study. The tree was constructed using the maximum likelihood method (1,000 bootstraps).

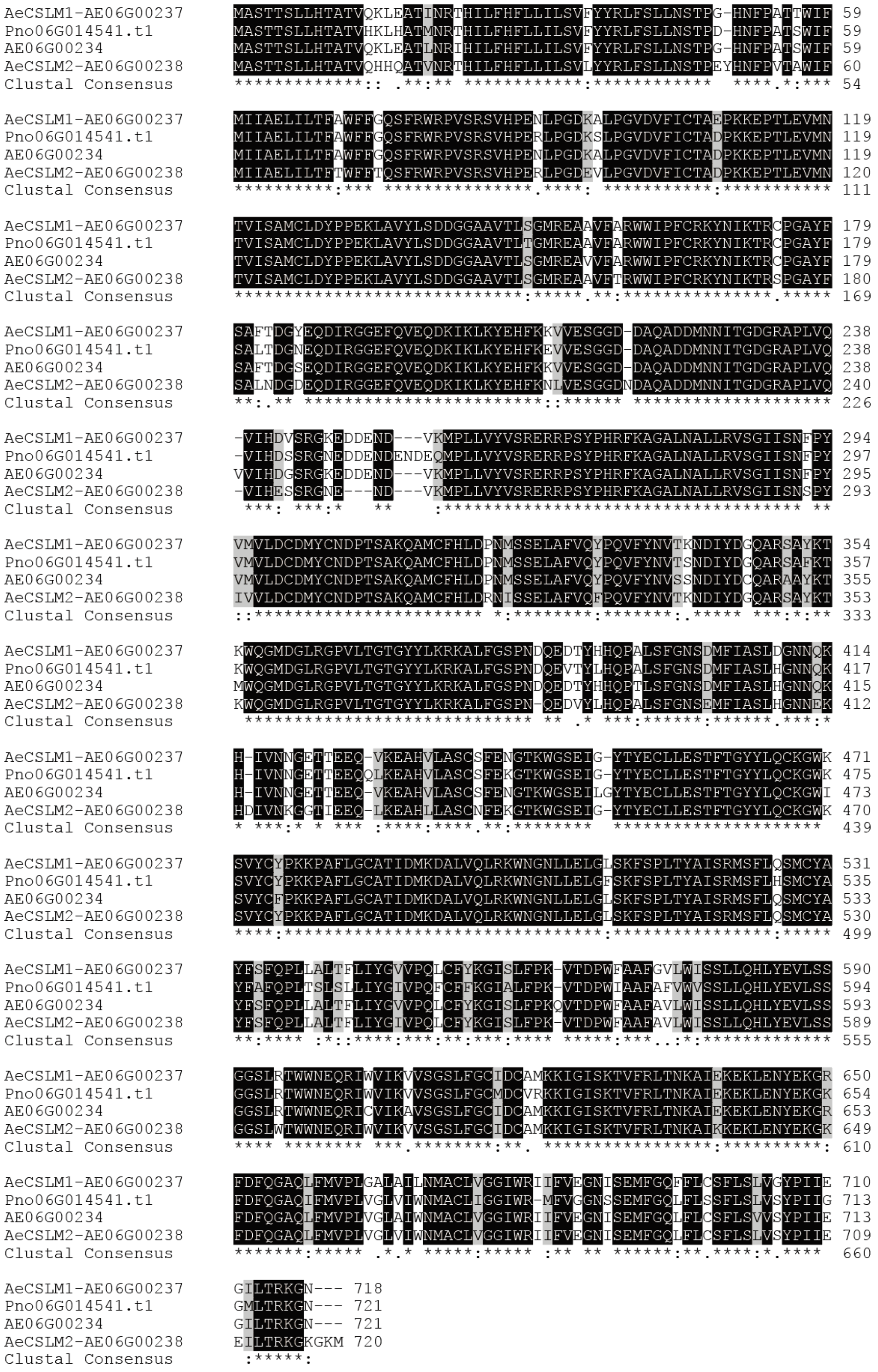

Supplementary Figure 32. Alignment of amino acid sequences of three AeCSLM members duplicated on chromosome 06 of *A. elata* the collinear CSLM enzyme in *P. notoginseng*.

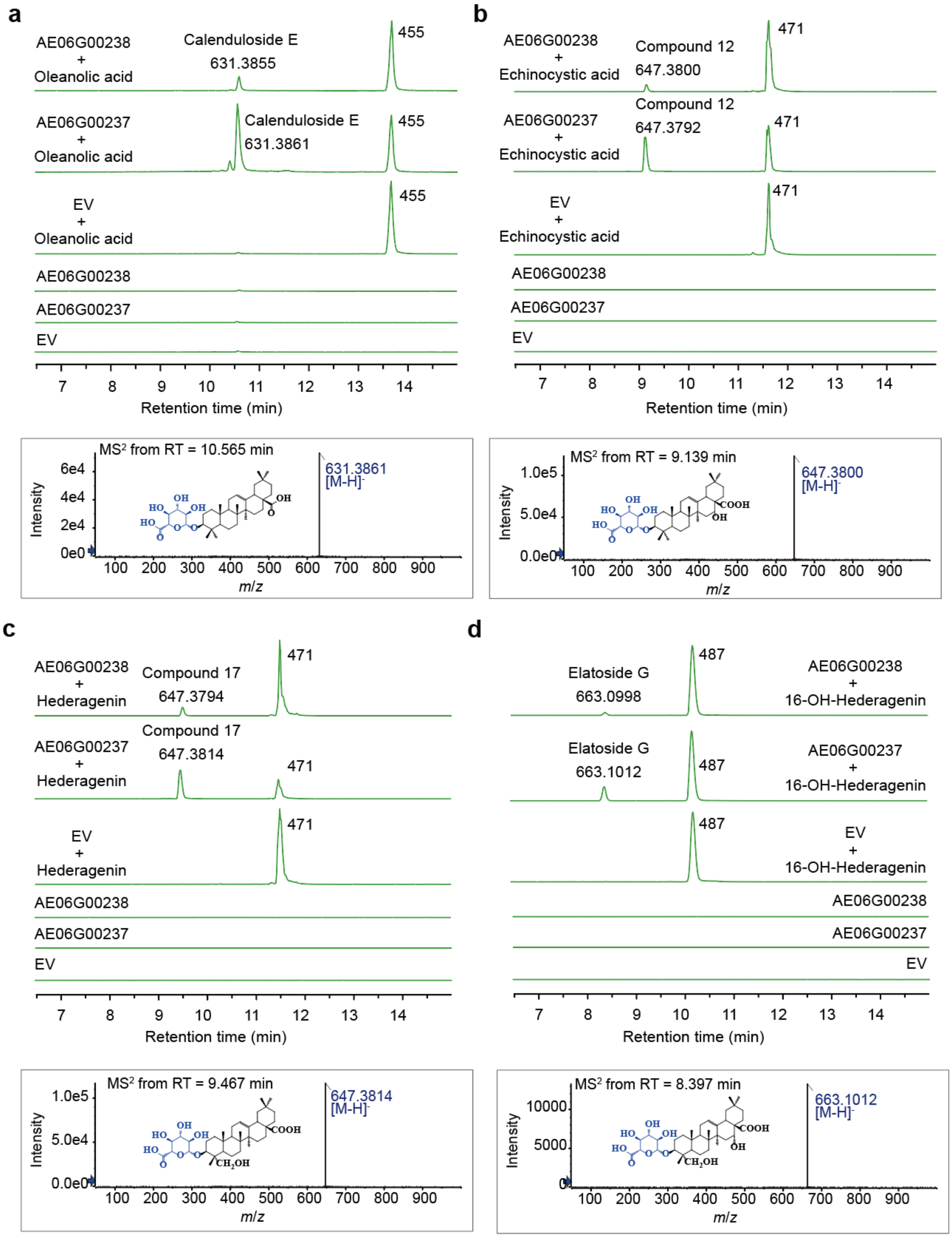

Supplementary Figure 33. Identification of AeCSL function. Overlays of LC–MS chromatograms obtained by extract ions chromatogram (EIC) of the theoretical *m*/*z* values of the compounds of interest. Chromatograms of monoglucuronides produced *in vivo* by AE06G00237 (AeCSLM1) or AE06G00238 (AeCSLM2), Oleanolic acid (**a**), Echinocystic acid (**b**), Hederagenin (**c**) and 16-OH-Hederagenin (**d**) as the substrates. The strain containing the empty vector (EV) with substrates served as the

negative control, and all strains without substrates served as blank controls. The MS/MS spectra of the product of AE06G00237 with each substrate is shown in the block; the observed fragmentation of each spectrum is shown in blue.

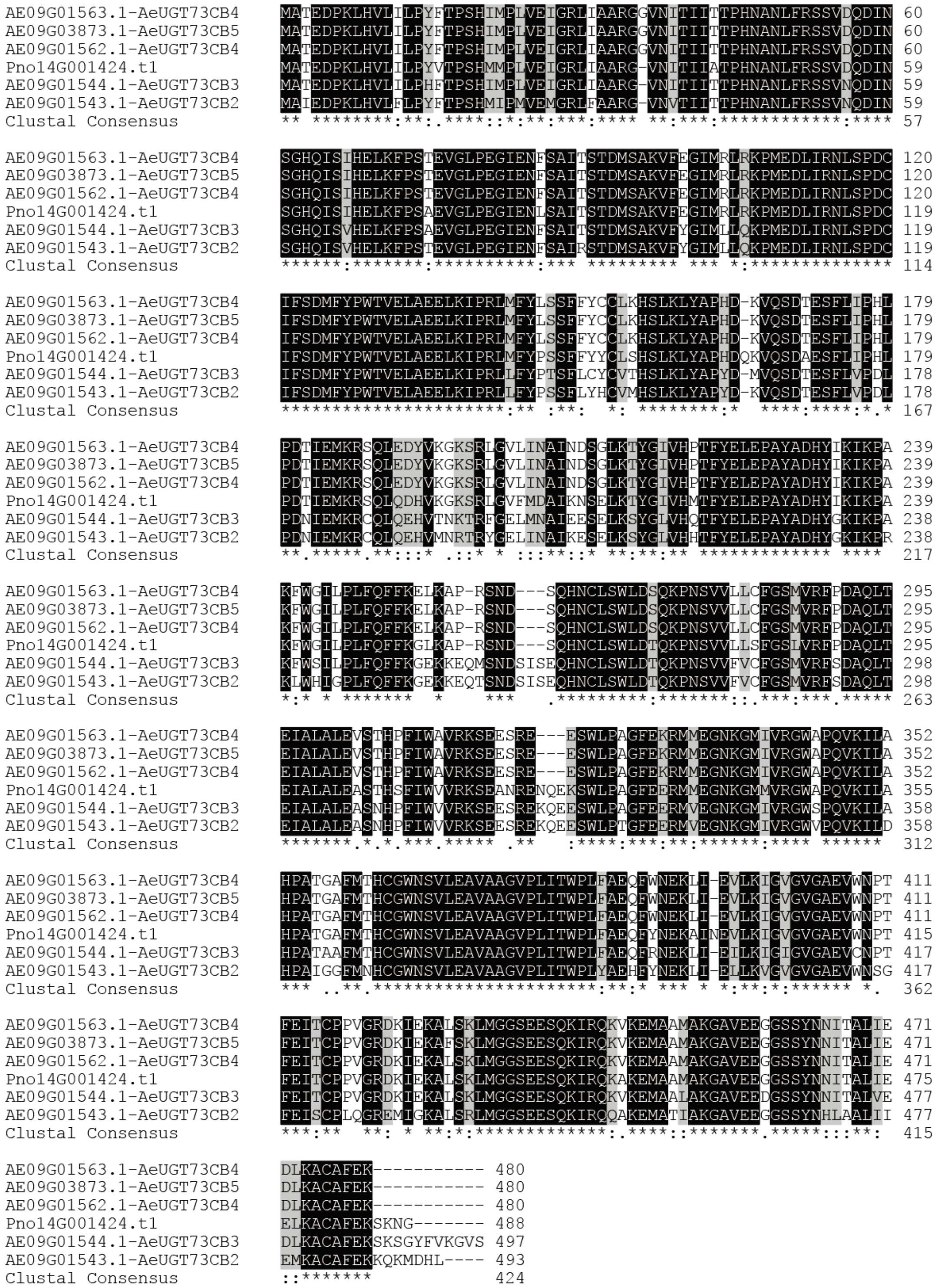

Supplementary Figure 34. Alignment of amino acid sequences of five AeUGT73 members duplicated in chromosome 09 of *A. elata* and the collinear UGT enzyme in *P. notoginseng*.

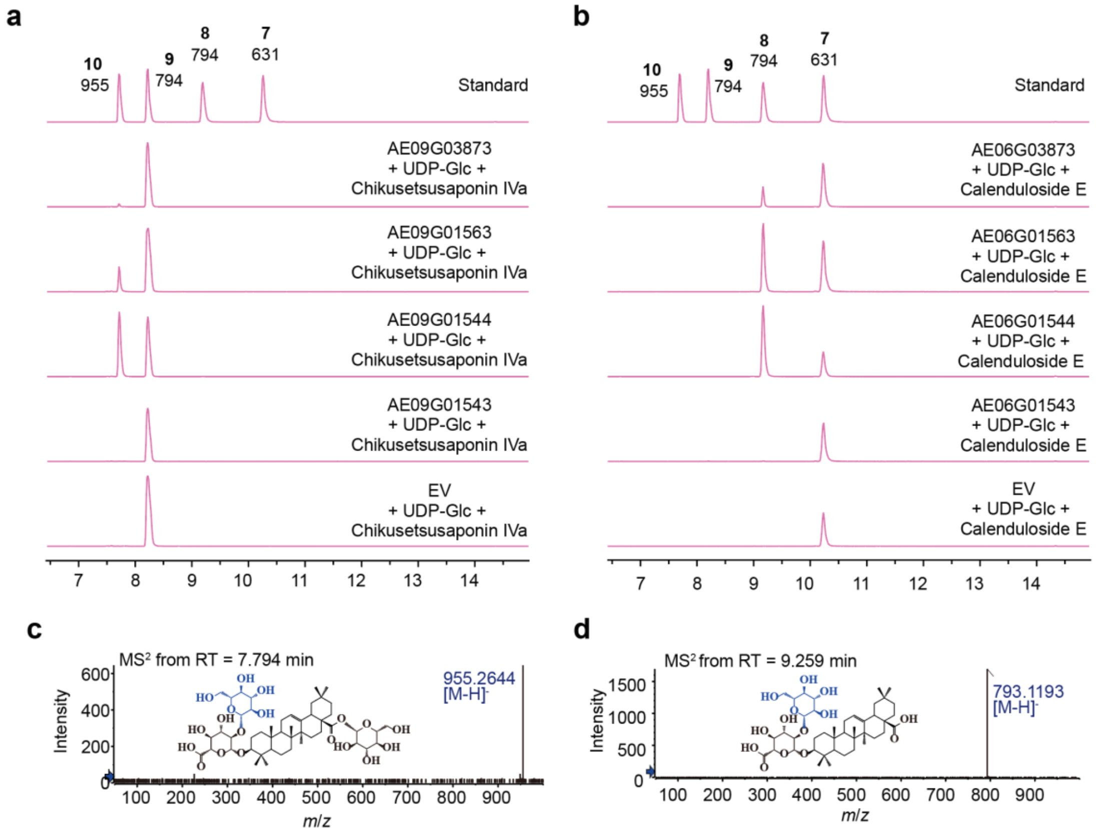

Supplementary Figure 35. ***In vitro*** **enzymatic function identification of three AeUGT73CB members.** Overlays of LC–MS chromatograms obtained by extract ions chromatogram (EIC) of the theoretical *m*/*z* values of the compounds of interest. Chromatograms of alarosides produced *in vitro* by AE09G01543 (AeUGT73CB2), AE09G01544 (AeUGT73CB3), AE09G01563 (AeUGT73CB4) and AE09G03873 (AeUGT73CB5). Chikusetsusaponin IVa (**a**) and Calenduloside E (**b**) were used as substrates for glycosylation. The crude enzyme extracted from the strain containing the empty vector (EV) with substrates served as the negative control. The MS/MS spectra of the product of AE09G01544 with Chikusetsusaponin IVa (**c**) and Calenduloside E (**d**) is shown in the block; the observed fragmentation of each spectrum is marked in blue. The structure of the product at the same retention time as the standard is shown in the blank space of the MS spectra. The number of standards in **a** and **b** were represent Calenduloside E (7), Zingibroside R1 (8), Chikusetsusaponin IVa (9) and Ginsenoside Ro (10).

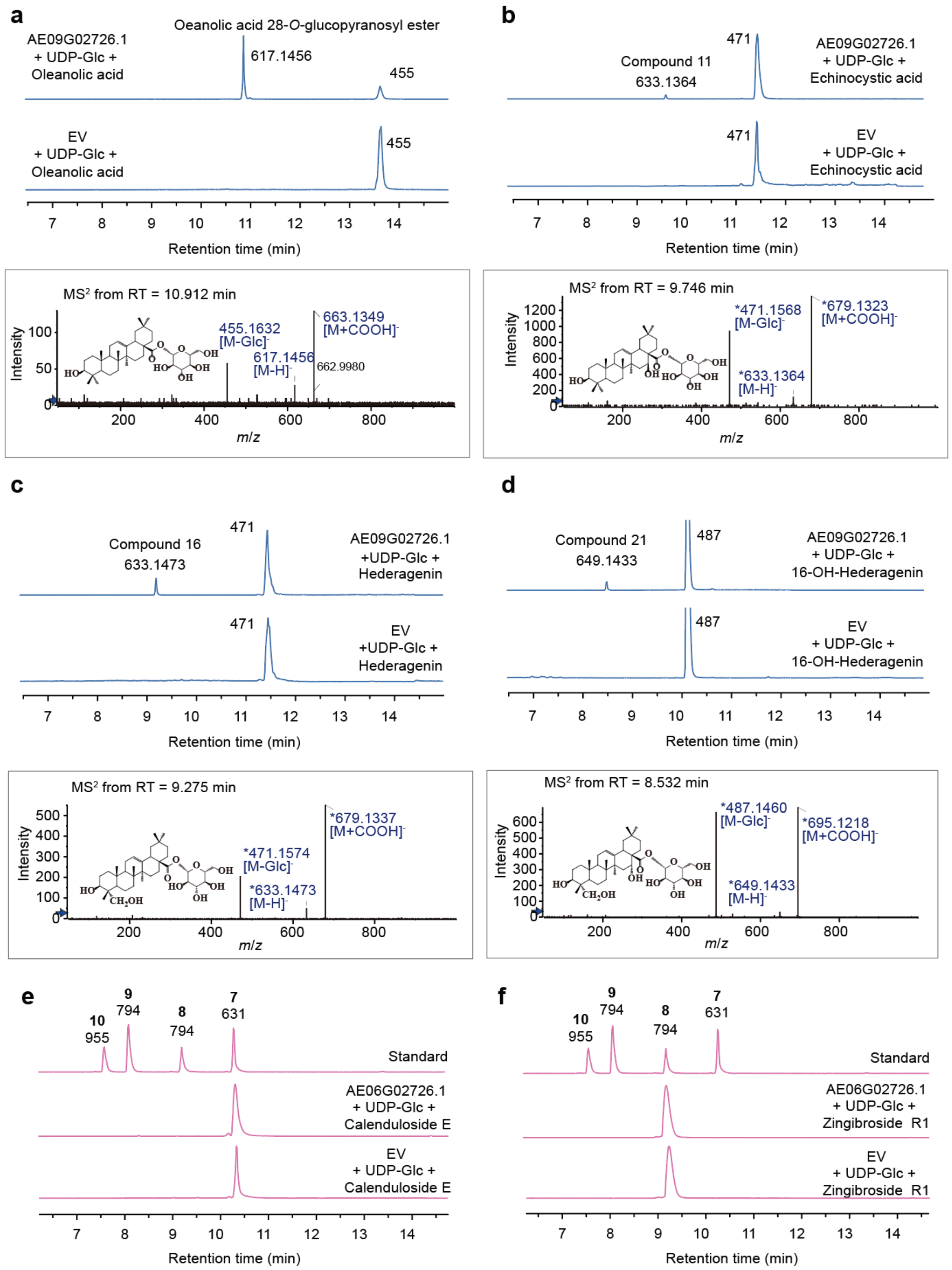

Supplementary Figure 36. *In vitro* enzymatic function identification of AeUGT73AD2. Overlays of LC–MS chromatograms obtained by extract ions chromatogram (EIC) of the theoretical *m/z* values of the compounds of interest. Chromatograms of alarosides produced *in vitro* by AE09G02726 (AeUGT73AD2) by catalyzing Oleanolic acid (**a**), Echinocystic acid (**b**), Hederagenin (**c**), 16-OH-Hederagenin (**d**) Calenduloside E (**e**) and Chikusetsusaponin IVa (**f**). The

crude enzyme extracted from the strain containing the empty vector (EV) with substrates served as the negative control. The MS/MS spectra of the product of AE09G02726 with each substrate is shown in the block; the observed fragmentation of each spectra is marked in blue color. The structure of each product is shown in the blank space of each MS spectrum. The number above the respective peaks in the spectra for the standards in **e** and **f** represent Calenduloside E (7), Zingibroside R1 (8), Chikusetsusaponin IVa (9) and Ginsenoside Ro (10).

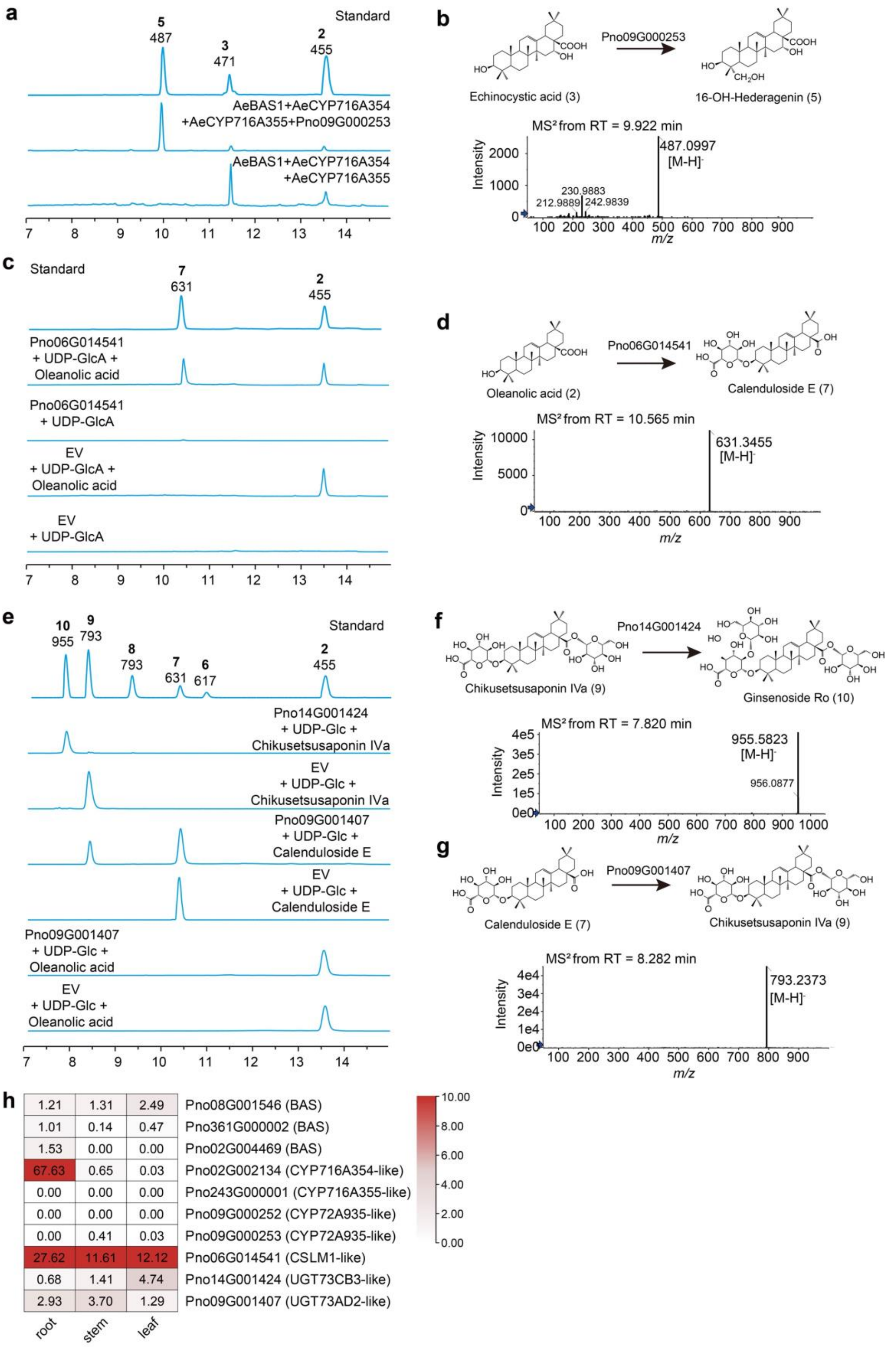

Supplementary Figure 37. *In vitro* and *in vivo* enzymatic functions identification of colinear enzymes from *P. notoginseng* in Figure 5. Overlays of LC–MS

chromatograms obtained by extract ions chromatogram (EIC) of the theoretical *m*/*z* values of the compounds of interest. **a**. Extract ion chromatogram overlay of the yeast strains expressing the *AeBAS1*, *AeCYP716A354*, *AeCYP716A355*, and *Pno09G000253* or without the Pno09G000253 as control. Peaks potentially corresponding to saponins are labeled with the *m*/*z* value of oleanolic acid (2, *m*/*z* 455), echinocystic acid (3, *m*/*z* 471), and 16-OH-hederagenin (5, *m*/*z* 487), and the MS/MS spectra are shown in **b**. **c**. Chromatograms of monoglucuronides produced *in vivo* by Pno06G014541 using oleanolic acid as the substrate. The strain containing the empty vector (EV) with substrate served as the negative control, and strain without substrate served as blank control. Peaks potentially corresponding to saponins are labeled with the *m*/*z* value of oleanolic acid (2, *m*/*z* 455) and calenduloside E (7, *m*/*z* 631), the MS/MS spectra of the product of Pno06G014541 with oleanolic acid as substrate is shown in **d**. **e**. Chromatograms of alarosides produced *in vitro* by Pno09G001407 and Pno14G001424 by catalysis of chikusetsusaponin IVa (**f**) and calenduloside E (**g**), respectively. The crude enzyme extracted from the strain containing the empty vector (EV) with substrates served as the negative control. **h**. Expression levels (FPKM) of colinear genes from *P. notoginseng* in root, stem, and leaf. Three biological replicates were used for RNA-seq analyses of different tissues from *P. notoginseng*. We averaged the FPKM values from replicate samples for drawing the heatmap.

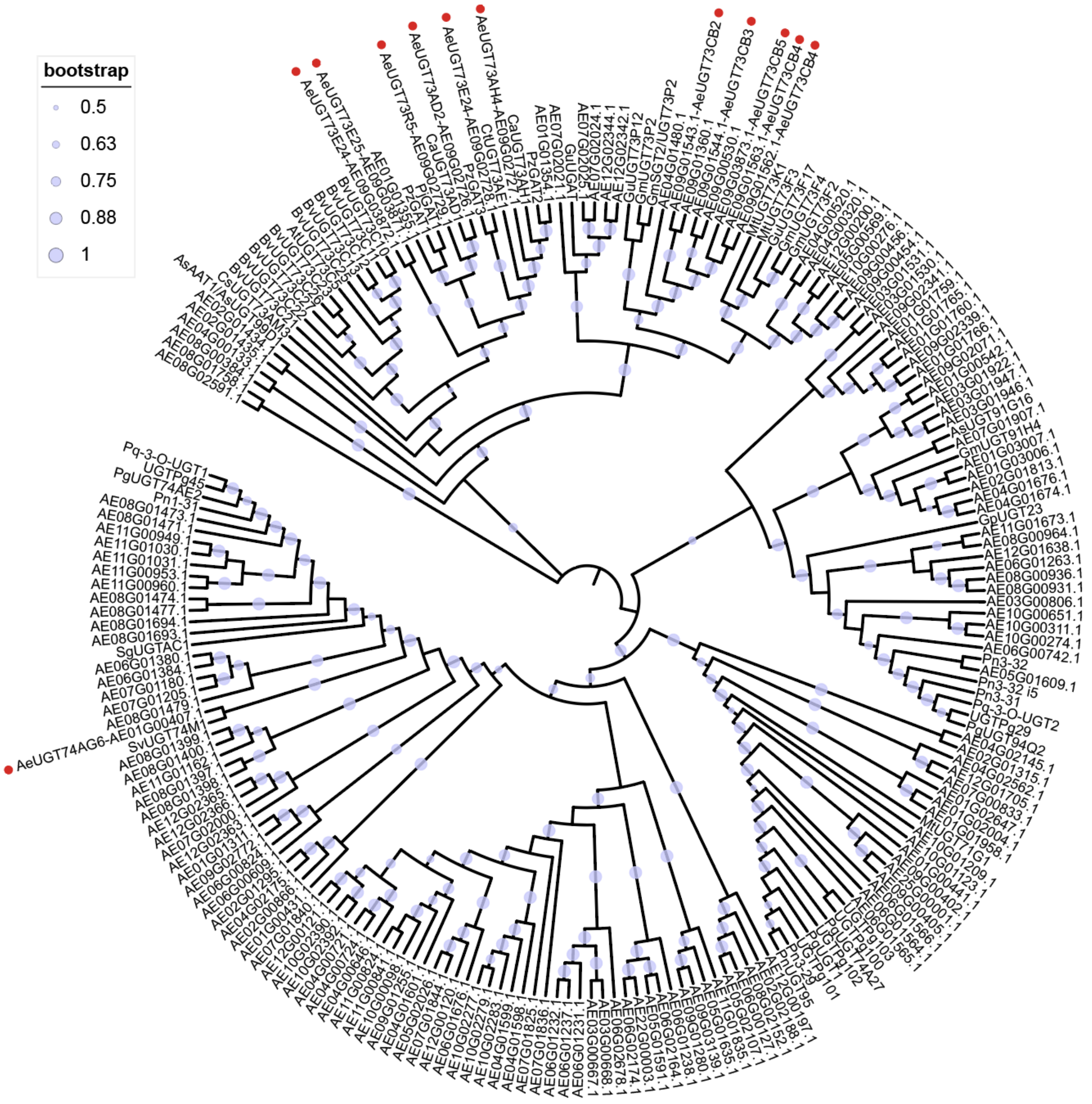

Supplementary Figure 38. Phylogenetic tree of UGT gene family in different species. Red dots indicate the tandem repeat genes analyzed in this study. The tree was constructed using the maximum likelihood method (1,000 bootstraps).

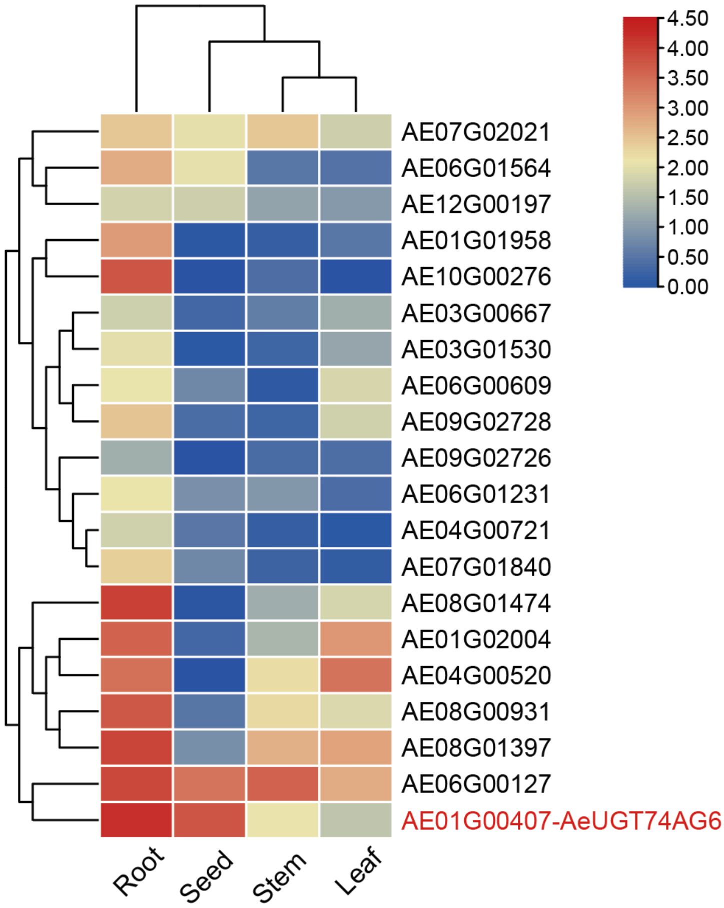

Supplementary Figure 39. Expression pattern (FPKM) of 20 *UGT* genes in leaf, stem, root and seed of *A. elata*. The heatmap is based on hierarchical clustering of expression profiles, and expression levels were normalized across conditions. Three biological replicates were used for RNA-seq analyses of different tissues from *A. elata*. We averaged the FPKM values from replicate samples for drawing the heatmap. Source data are provided as a Source Data file.

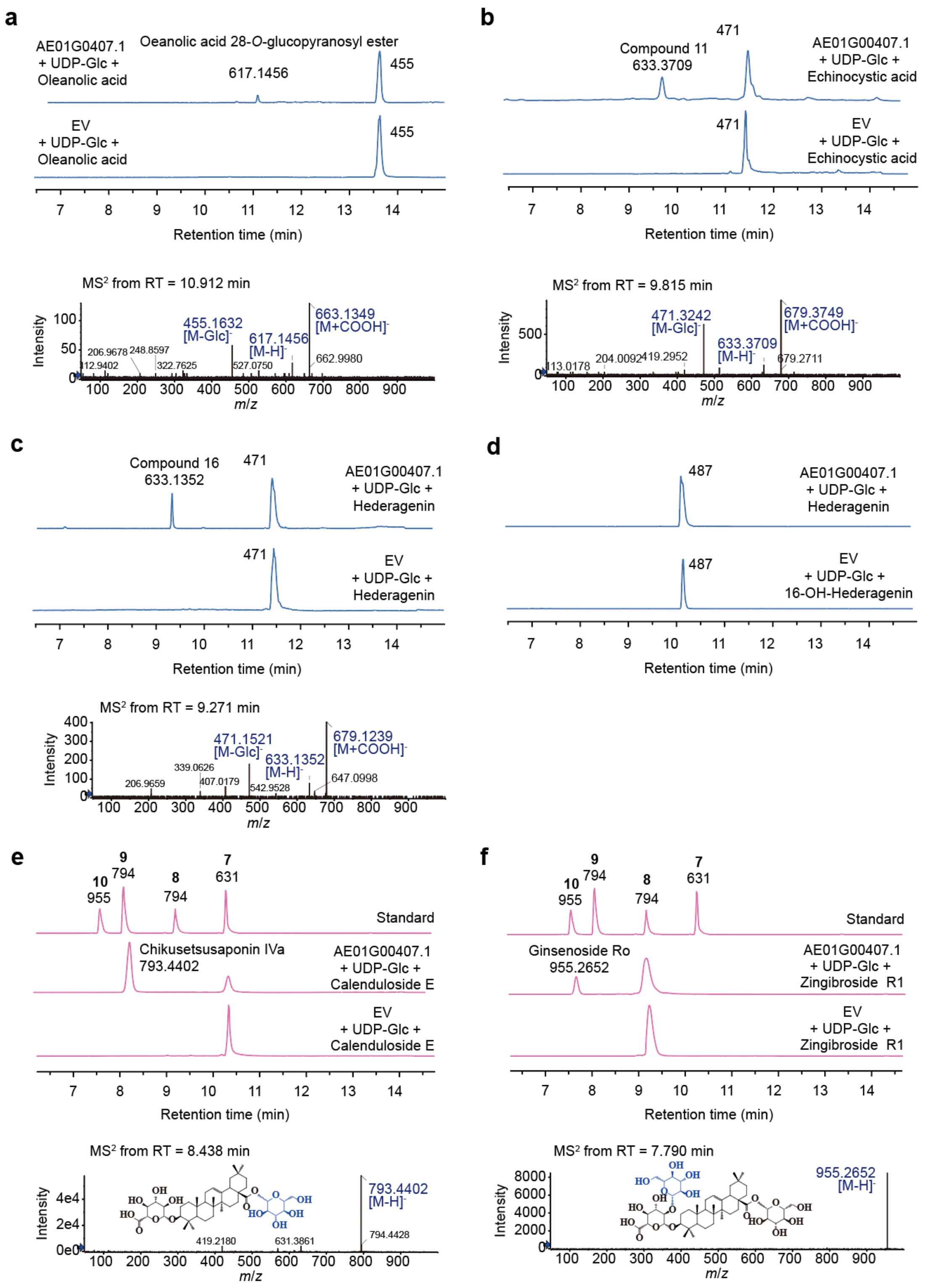

Supplementary Figure 40. *In vitro* enzymatic function identification of AeUGT74AG6. Overlays of LC–MS chromatograms obtained by extract ions chromatogram (EIC) of the theoretical *m/z* values of the compounds of interest. Chromatograms of alarosides produced *in vitro* by AE01G00407 (AeUGT74AG6) by catalysis of Oleanolic acid (**a**), Echinocystic acid (**b**), Hederagenin (**c**),

16-OH-Hederagenin (**d**) Calenduloside E (**e**) and Chikusetsusaponin IVa (**f**). The crude enzyme extracted from the strain containing the empty vector (EV) with substrates served as the negative control. The MS/MS spectrum of the reaction product of AE01G00407 with each substrate is shown in the block; the observed fragmentation of each spectrum is marked in blue. The structure of the product is shown in the blank space of each MS spectrum. The number above the respective peaks in the spectra for the standards in **e** and **f** represent Calenduloside E (7), Zingibroside R1 (8), Chikusetsusaponin IVa (9) and Ginsenoside Ro (10).

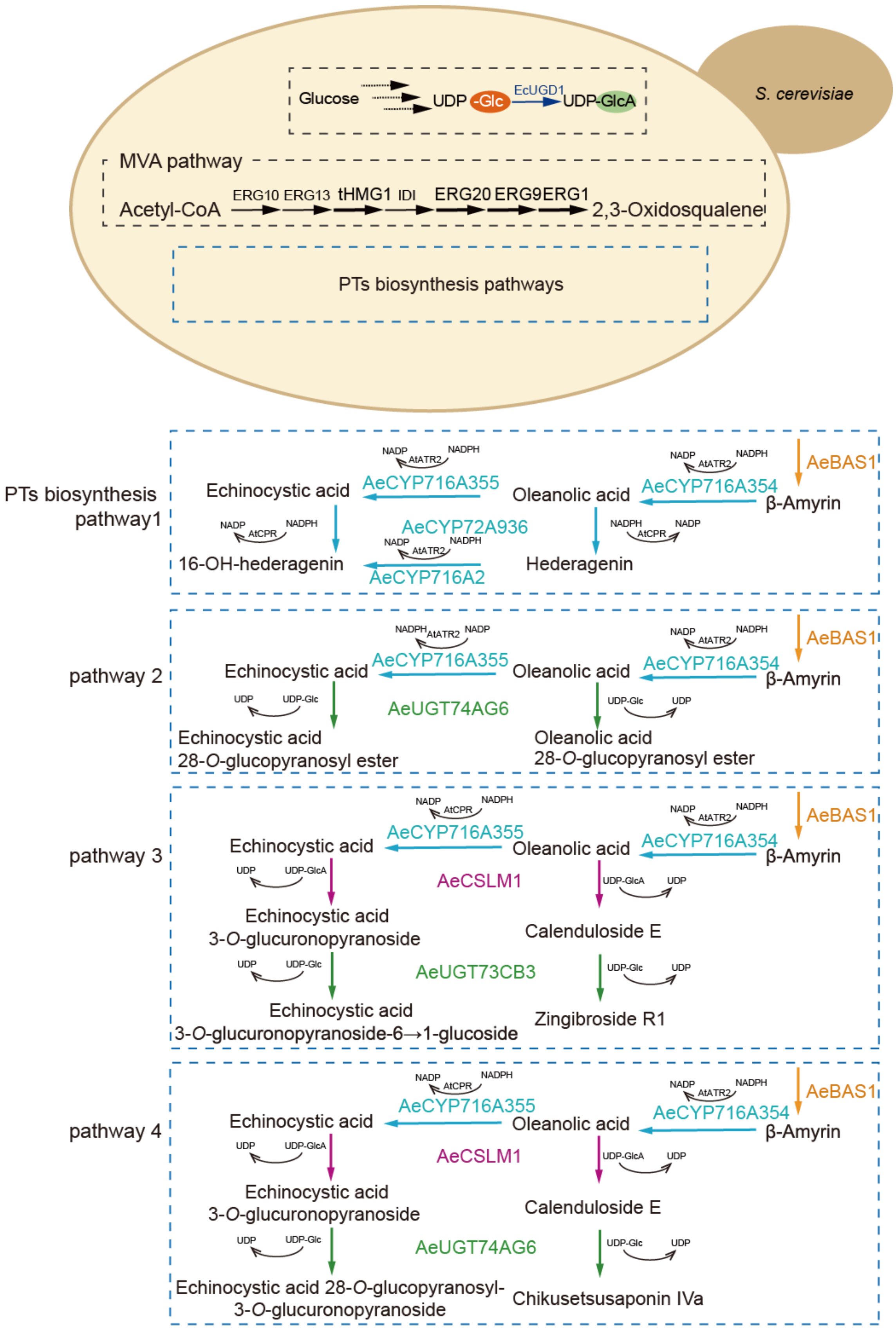

Supplementary Figure 41. Schematic diagram of the yeast engineered for *de novo* biosynthesis of aralosides. Strain WEA overexpressed the MVA and early sterol

pathway genes *tHMG1*, *ERG20*, *ERG9* and *ERG1* as the black-dashed box show. The UDP-glucose 6-dehydrogenase 1 (UGD1) from *E. coli* was expressed in the yeast to enable synthesis of UDP-glucuronic acid. The biosynthetic pathways for four pentacyclic triterpenes were introduced into yeast strain WEA as shown in the blue-dashed box. Yellow arrows indicate a cyclization reaction catalyzed by β-amyrin synthase (BAS); blue arrows indicate an oxidation reaction catalyzed by cytochrome P450 (P450s); purple arrows indicate a glycosylation reaction catalyzed by cellulose synthase-like (CSL); green arrows indicate glycosylation reactions catalyzed by UDP-dependent glycosyltransferases (UGTs). AtCPR1 supplies the cofactor NADPH for P450.

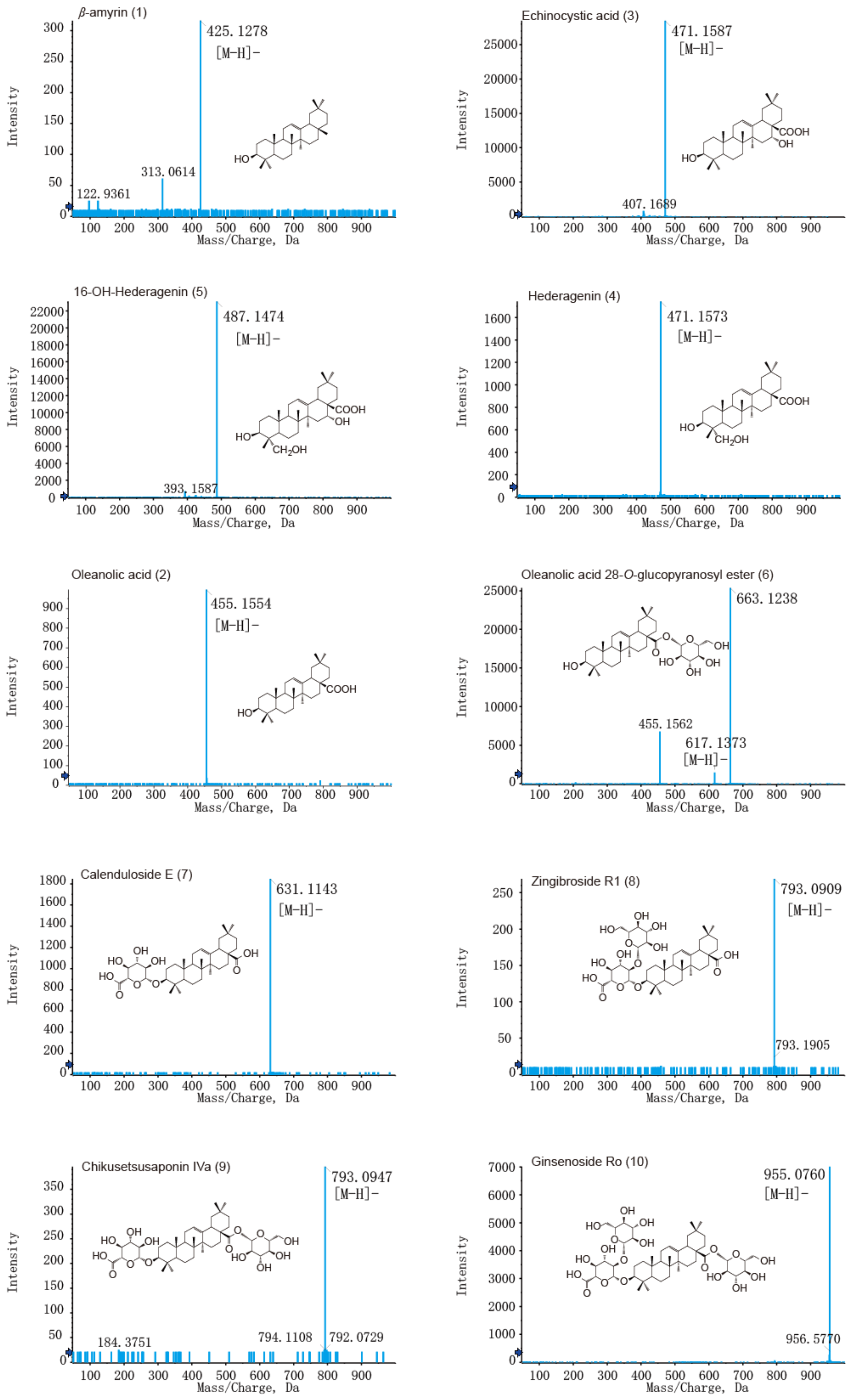

Supplementary Figure 42. MS/MS fragmentation of standard from compound 1 to 10.

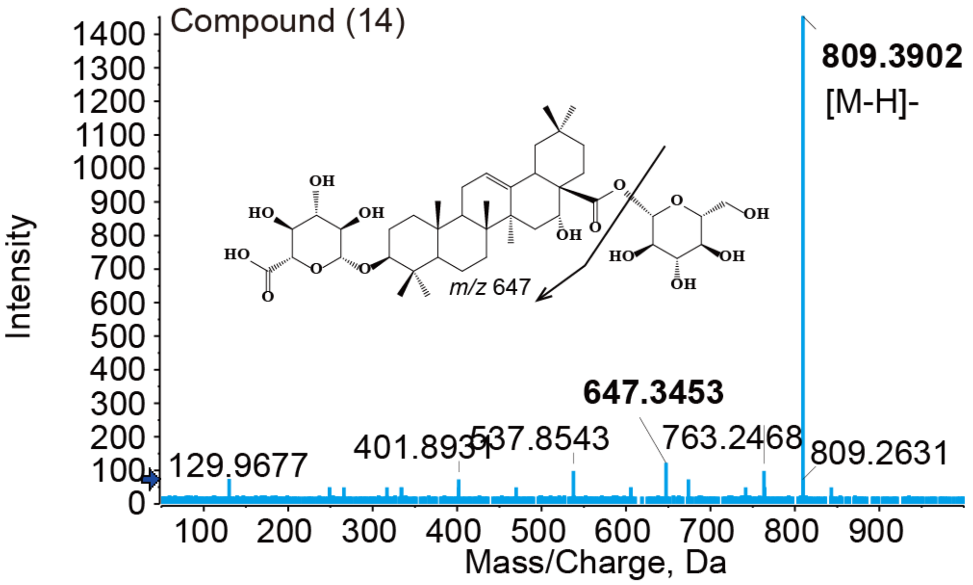

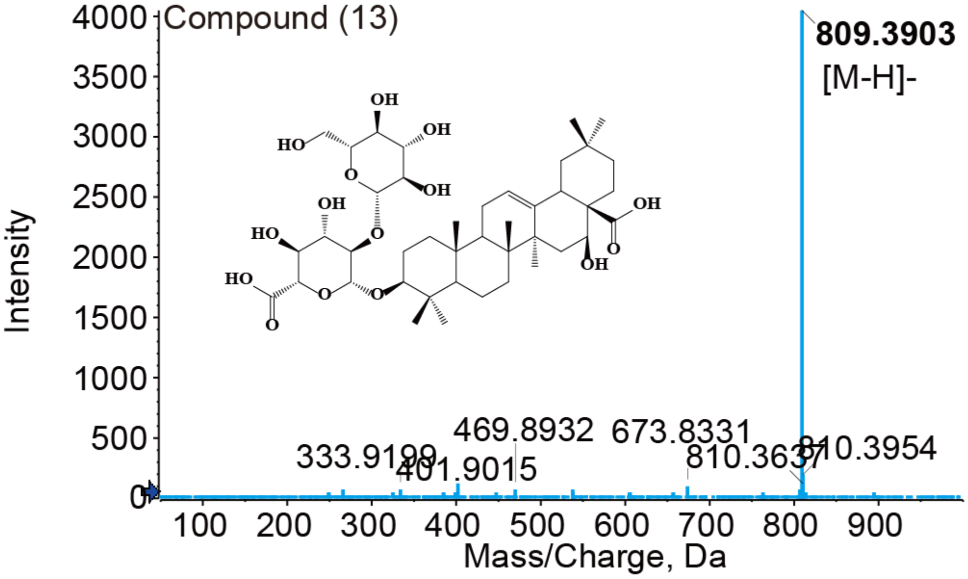

Supplementary Figure 43. MS/MS fragmentation of product compound 14 and 13 from *de novo* biosynthesis in yeast.

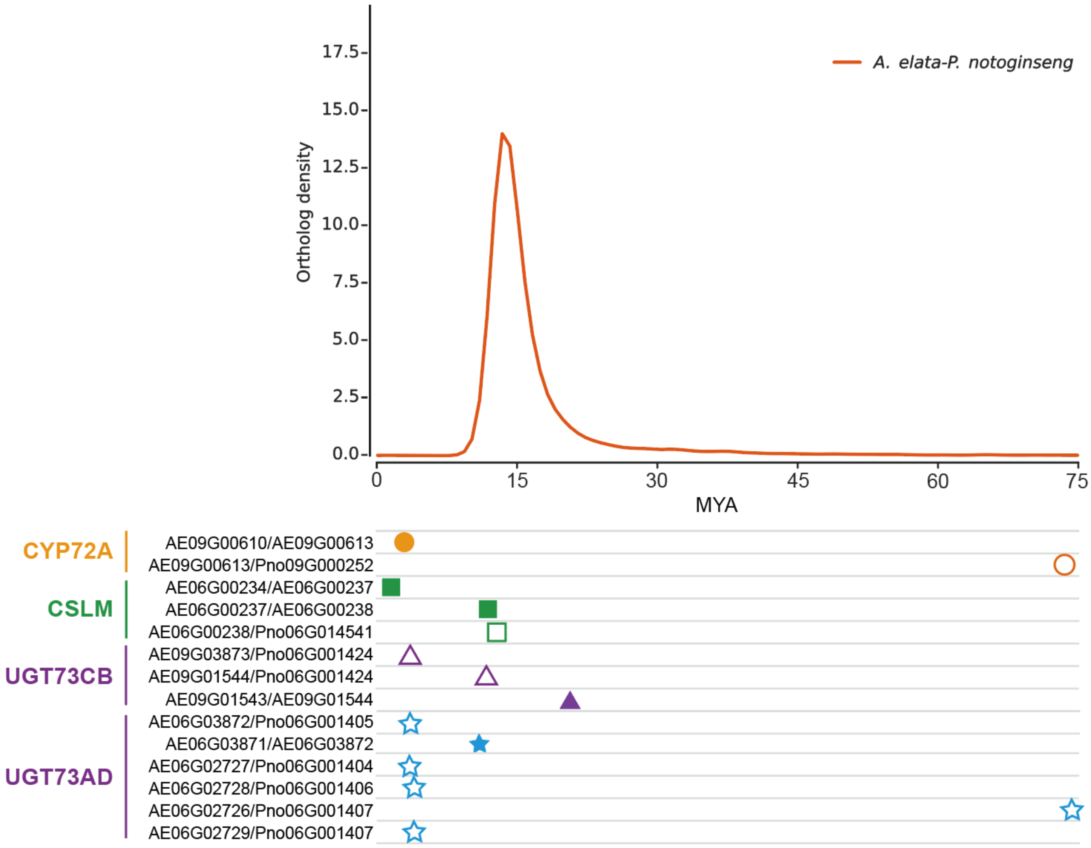

Supplementary Figure 44. *K*s analysis of divergence time of the gene pairs involved in araloside biosynthesis. Open symbols indicate orthologous gene pairs; solid symbols indicate paralogous gene pairs. The calculated *K*s value was converted to divergence time according to T=*K*s/2r. Source data are provided as a Source Data file.

Supplementary Table 1. Sequencing statistics using PacBio.

ID	ZMWNUM	Total no. bases (Gb)	Total no. reads	Average length (bp)	Maximum length (bp)	N50 length (bp)
m64048_201026_201631	6,371,156	97.88	7,556,167	12,954.19	232,237	20,279

Supplementary Table 2. Summary of *A. elata* genome assembly.

Statistic	Contig		Scaffold	
	**Size (bp)**	**Number**	**Size (bp)**	**Number**
N90	245,166	936	245,166	936
N80	468,306	634	468,306	634
N70	706,971	454	706,971	454
N60	953,751	326	953,751	326
N50	1,195,804	229	1,195,804	229
Longest	17,272,275	-	17,272,275	-
Total length	1,046,177,497	-	1,046,177,497	-
Total no. (≥100 bp)	-	2,035	-	2,035
Total no. (≥2000 bp)	-	2,035	-	2,035
GC rate	0.355	-	0.355	-

Supplementary Table 3. HiC-assisted assembly results of *A. elata*.

Statistic	Contig		Scaffold	
	**Size (bp)**	**Number**	**Size (bp)**	**Number**
N90	245,166	936	73,159,195	11
N80	468,306	634	79,603,388	10
N70	706,971	454	83,455,037	8
N60	953,751	326	83,843,920	7
N50	1,195,804	229	85,555,957	6
Longest	17,272,275	-	110,444,823	-
Total Length	1,046,177,497	-	1,047,181,497	-
Total no. (≥100 bp)	-	2,035	-	27
Total no. (≥2000 bp)	-	2,035	-	27
GC Rate	0.355	-	0.354	-

Supplementary Table 4. Chromosome length of *A. elata* genome.

Chromosome ID	Length (bp)
Chr01	81,024,418
Chr02	83,455,037
Chr03	73,159,195
Chr04	85,555,957
Chr05	79,603,388
Chr06	108,756,462
Chr07	94,211,576
Chr08	110,444,823
Chr09	88,419,863
Chr10	83,843,920
Chr11	96,582,347
Chr12	52,958,921

Supplementary Table 5. Basic statistical results of gene structure prediction of *A. elata* genome.

Methods	Gene set	Number	Average gene length (bp)	Average CDS length (bp)	Average exon per gene	Average exon length (bp)	Average intron length (bp)
De novo	**Augustus**	124631	2,965.86	881.5	3.43	256.7	856.37
	**Snap**	216080	3,142.59	597.19	3.43	173.87	1045.51
**Homolog**	***Panax ginseng***	647596	1,940.85	664.86	2.13	312.1	1128.96
	Panax notoginseng	365050	1,850.86	621.37	2.13	291.75	1088.3
	Apium graveolens	132105	3,036.52	866.6	3.2	270.54	984.91
	Coriandrum sativum	243978	2,791.28	771.28	2.73	282.78	1169.3
	Mikania micrantha	237906	2,541.39	799.67	2.74	286.88	1013.42
	Arabidopsis thaliana	97517	3,559.40	942.32	3.48	270.88	1055.81
**RNA-seq**	-	231679	3,151.29	1,323.36	2.91	454.1	954.91
**Maker**	-	35,042	6,216.05	1,311.73	5.6	234.37	869.2

Supplementary Table 6. Functional annotation results for predicted proteins in *A. elata*.

Values	Total	Nr	Swissprot	KEGG	KOG	TrEMBL	Interpro	GO	Overall
**Number**	35,042	33,534	25,102	25,164	24,336	33,270	30,446	17,639	33,647
**Percentage**	-	95.70%	71.63%	71.81%	69.45%	94.94%	86.88%	50.34%	96.02%

Supplementary Table 7. Statistics results of repeat sequences.

Type	Repeat size(bp)	Percentage of genome
**TRF**	50,161,845	4.794774
**RepeatMasker**	209,319,084	20.007990
**RepeatProteinMask**	183,039,992	17.496074
De novo	651,791,063	62.302149
**Total**	686,334,870	65.604056

Supplementary Table 8. Classified repeat sequences of *A. elata* genome.

Type	RepBase TEs		TE proteins		*De novo* predicted		Combined TEs	
	Length (bp)	Percentage of genome	Length (bp)	Percentage of genome	Length (bp)	Percentage of genome	Length (bp)	Percentage of genome
DNA	11131932	1.064058	2335546	0.223246	32926290	3.147295	41132760	3.931719
LINE	1369132	0.13087	483709	0.046236	3536077	0.338	5053025	0.482999
SINE	27997	0.002676	0	0	29386	0.002809	57383	0.005485
LTR	198036439	18.92953	180222882	17.2268	616564152	58.93495	633850262	60.58726
Other	3525	0.000337	0	0	0	0	3525	0.000337
Unknown	0	0	0	0	2725876	0.260556	2725876	0.260556
Total	209319084	20.00799	183039992	17.49607	650496686	62.17843	672267068	64.25937

Supplementary Table 9. Statistics of repeat sequence in *A. elata* genome.

Note: Total is the result obtained by the above methods and the non-redundant result after removing the overlap between them.

Type	Length (bp)	Percentage of genome
DNA	41132760	3.93172
LINE	5053025	0.48300
SINE	57383	0.00549
LTR/Copia	186944630	17.86930
LTR/Gypsy	457833921	43.76255
LTR/other	36358948	3.47542
Satellite	225668	0.02157
Simple repeat	1068709	0.10215
Other	3525	0.00034
Unknown	2725876	0.26056
Tandam repeat	50161845	4.79477
Total	686334870	65.60406

Supplementary Table 10. BUSCO assessment of *A. elata* genome assembly and annotation.

Type	Genome		Protein	
	**Count**	**Percentage**	**Count**	**Percentage**
Complete BUSCOs (C)	1,594	98.8%	1,583	98.1%
Complete and single-copy BUSCOs (S)	1,414	87.6%	1,411	87.4%
Complete and duplicated BUSCOs (D)	180	11.2%	172	10.7%
Fragmented BUSCOs (F)	5	0.3%	17	1.1%
Missing BUSCOs (M)	15	0.9%	14	0.8%
Total BUSCO groups searched	1,614	-	1,375	-

Supplementary Table 11. Clustering results of gene families in 16 species.

Species	No. of genes number	No. of genes in families	Unclustered genes	No. of families	No. of unique families	Mean no. of genes per family
Aralia elata	35,042	29,249	5,790	15,408	507	1.90
Eleutherococcus senticosus	36,372	30,888	5,484	13,960	309	2.21
Amborella trichopoda	26,846	19,244	7,602	12,325	1,050	1.56
Oryza sativa	42,173	30,586	11,587	16,725	1,555	1.83
Brachypodium distachyon	34,309	25,877	8,432	15,913	782	1.63
Populus trichocarpa	42,945	33,769	9,176	14,917	957	2.26
Arabidopsis thaliana	27,342	23,545	3,797	12,991	692	1.81
Vitis vinifera	31,315	23,969	7,346	14,536	851	1.65
Daucus carota	32,106	26,424	5,682	13,956	911	1.89
Panax ginseng	59,352	44,302	15,050	17,412	1,464	2.54
Panax notoginseng	41,234	33,610	7,624	14,570	852	2.31
Eucalyptus grandis	36,349	29,025	7,324	13,729	780	2.11
Prunus persica	26,873	22,670	4,203	14,248	452	1.59
Solanum lycopersicum	35,343	25,931	9,412	14,401	965	1.8
Coffea canephora	25,574	21,056	4,518	13,620	518	1.55
Helianthus annuus	52,243	39,185	13,058	15,164	2,220	2.58

Supplementary Table 12. Kinetic parameters for UGTs using different substrates.

UGT	Substrates	K_m (μM)	K_{cat} (min^{-1})	k_{cat}/K_m (min^{-1} mM^{-1})
AeUGT73AD2	oleanolic acid	43.04 ± 9.17	0.32	7.53 ± 0.37
AeUGT73CB3	calenduloside E	51.76 ±2.10	5.78	1.86 ± 0.01
	chikusetsusaponin IVa	640.24 ± 4.60	2.87	4.49 ± 0.12
AeUGT74AG6	oleanolic acid	40.29 ± 2.49	0.08	1.95 ± 0.04
	calenduloside E	149.54 ± 7.70	2.57	17.18 ± 0.06
	zingibroside R1	48.78 ± 3.04	2.23	45.85 ± 1.83

Kinetic parameters were calculated by fitting the initial velocity data to the Michaelis-Menten equation by nonlinear regression analysis. For determination of relative K_{cat}, the product was quantified relative to the amount of product formed by the 1 μM purified enzyme at 0.5 mM UDP-Glc for 1 h. The experiment was performed as described in Supplementary Notes 2.

Supplementary Table 13. Accession numbers of enzymes used in this study.

Enzyme	Accession
AeBAS1	OK094504
AeBAS2	OK094505
AeAS3	OM641779
AeCYP716A354	OK094506
AeCYP716A355	OK094507
AeCYP716A356	OM641780
AeCYP716A357	OM641781
AeCYP72A935	OK094508
AeCYP72A936	OK094509
AeCYP72A937	OM641782
PnCYP72A938	OM641783
AeCSLM1	OK094510
AeCSLM2	OK094511
Pno06G014541	OM641784
AeUGT73AD2	OK094512
AeUGT74AG6	OK094513
AeUGT73CB3	OK094514
AeUGT73CB2	OK094515
AeUGT73CB4	OK094516
AeUGT73CB5	OK094517
Pno14G001424	OM641785
Pno09G001407	OM641786

Supplementary Note 1. Whole-genome duplication events verification among *A. elata*, *D. carota*, and *V. vinifera* genomes.

In order to investigate the whole-genome duplication events in Apiales, we further analyzed the genome synteny and *K*s values of paralogs and orthologs among *A. elata*, *D. carota*, and *V. vinifera* genomes.

First, the intra-genomic collinearity showed that the *A. elata* experienced only one WGD event (Araliaceae-specific WGD) after the core-eudicot whole-genome triplication (WGT, or *γ* event) (Supplementary Figure 12). In contrast, the *D. carota* underwent two rounds of polyploidization (Dc-beta and Dc-alpha genome duplications) (Supplementary Figure 13). The median Ks value of paralog gene pairs on synteny blocks from *A. elata* genome was about 0.3, while the Dc-beta and Dc-alpha corresponding to the median Ks values of ~1.0 and ~0.6, indicating these WGD events may not occurred close in time (Supplementary Figures 12 and 13).

Second, the well-characterized grape (*V. vinifera*) genome, which is relatively stable genome and is likely not affected by any polyploidization event after the *γ* event, was used as a reference for inter-genomic dotplot comparison with *A. elata* and *D. carota*. The ratios of the best-matched orthologous regions between two species (*A. elata* and *D. carota*) and *V. vinifera* were 2:1 and 4:1, respectively (Supplementary Figures 14 and 15). This indicates that after divergence from grape, the *D. carota* experienced two additional whole genome duplication events, resulting in overlapping carrot homoeologous regions often up to 4× depth. If there had been an extra hexaploidization and tetraploidization event in carrot, as Iorizzo *et al*. reported[1],

assuming no DNA loss, we would expect a grape gene (or chromosomal region) to have six best-matched or orthologous carrot genes (chromosomal regions). Here, our findings indicated that the Dc-beta event was a tetraploidization instead of a triplication, which is consist with recent reports in Apiaceae[2-4].

Hence, assuming the Dc-beta event is shared with *Aralia elata*, we would expect that *A. elata* experienced two additional whole genome duplication events after the γ event and the ratio of the best-matched orthologous regions between *A. elata* and *D. carota* should be 2:2. However, we found a clear 2:4 collinearity relationship between *A. elata* and *D. carota* (Supplementary Figure 16). In summary, our results support that the Dc-beta WGD event (tetraploidization) occurred after the divergence of the Apiaceae and Araliaceae and that the independent WGD event shared in Araliaceae occurred after the Dc-beta WGD.

Supplementary references

1. Iorizzo M*, et al.* A high-quality carrot genome assembly provides new insights into carotenoid accumulation and asterid genome evolution. *Nature Genetics* **48**, 657-666 (2016).
2. Wang J*, et al.* Sequential Paleotetraploidization shaped the carrot genome. *BMC Plant Biol* **20**, 52 (2020).
3. Song X*, et al.* The celery genome sequence reveals sequential paleo-polyploidizations, karyotype evolution and resistance gene reduction in apiales. *Plant Biotechnol J* **19**, 731-744 (2021).
4. Song X*, et al.* Deciphering the high-quality genome sequence of coriander that causes controversial feelings. *Plant Biotechnol J* **18**, 1444-1456 (2020).
