# Supplementary material for: Deletion and tandem duplications of biosynthetic genes drive the diversity of triterpenoids in Aralia elata
Source: Nat Commun. 2022 Apr 25;13:2224. doi: 10.1038/s41467-022-29908-y (PMC9038795; doi:10.1038/s41467-022-29908-y)
Supplement: Supplementary file 3 — Description of Additional Supplementary Files [file 41467_2022_29908_MOESM3_ESM.pdf]

Description of Additional Supplementary Files

File Name: Supplementary Data 1
Description: The GeneBank ID of proteins used for phylogenetic tree construction in this study.

File Name: Supplementary Data 2
Description: Plasmids used in this study.

File Name: Supplementary Data 3
Description: Yeast strains constructed in this study.

File Name: Supplementary Data 4
Description: : Primers used in this study.
