# Supplementary material for: Deletion and tandem duplications of biosynthetic genes drive the diversity of triterpenoids in Aralia elata
Source: Nat Commun. 2022 Apr 25;13:2224. doi: 10.1038/s41467-022-29908-y (PMC9038795; doi:10.1038/s41467-022-29908-y)
Supplement: Supplementary file 8 — Reporting Summary [file 41467_2022_29908_MOESM8_ESM.pdf]

nature portfolio

Corresponding author(s): Yuhua Li

Last updated by author(s): Mar 23, 2022

Reporting Summary

Nature Portfolio wishes to improve the reproducibility of the work that we publish. This form provides structure for consistency and transparency in reporting. For further information on Nature Portfolio policies, see our Editorial Policies and the Editorial Policy Checklist.

Statistics

For all statistical analyses, confirm that the following items are present in the figure legend, table legend, main text, or Methods section.

n/a	Confirmed	
☐	☒	The exact sample size (*n*) for each experimental group/condition, given as a discrete number and unit of measurement
☐	☒	A statement on whether measurements were taken from distinct samples or whether the same sample was measured repeatedly
☒	☐	The statistical test(s) used AND whether they are one- or two-sided<br>*Only common tests should be described solely by name; describe more complex techniques in the Methods section.*
☒	☐	A description of all covariates tested
☐	☒	A description of any assumptions or corrections, such as tests of normality and adjustment for multiple comparisons
☐	☒	A full description of the statistical parameters including central tendency (e.g. means) or other basic estimates (e.g. regression coefficient) AND variation (e.g. standard deviation) or associated estimates of uncertainty (e.g. confidence intervals)
☒	☐	For null hypothesis testing, the test statistic (e.g. *F*, *t*, *r*) with confidence intervals, effect sizes, degrees of freedom and *P* value noted<br>*Give P values as exact values whenever suitable.*
☒	☐	For Bayesian analysis, information on the choice of priors and Markov chain Monte Carlo settings
☒	☐	For hierarchical and complex designs, identification of the appropriate level for tests and full reporting of outcomes
☒	☐	Estimates of effect sizes (e.g. Cohen's *d*, Pearson's *r*), indicating how they were calculated

Our web collection on statistics for biologists contains articles on many of the points above.

Software and code

Policy information about availability of computer code

Data collection	HPLC/MS: HPLC/TripleTOF 6600 system(AB Sciex), PeakView 2.2 (Sciex) , NMR: AscendTM 800 MHz spectrometer (Bruker)
Data analysis	K-mer analysis: SOAPnuke (v2.0.2), JELLYFISH (v.2.2.6), GenomeScope;<br>Genome assembly: Canu(v2.1), BWA (v0.7.17), Pilon (v1.23),SOAPnuke (v2.0.2), Juicer (v2.0), 3d-DNA (v 180922), Juicerbox (v1.11.08), BUSCO v3 (embrophyta_odb10);<br>Genome annotation: GeneWise (v2.4.1), AUGUSTUS (v3.2.1), SNAP (v2006-07-28), HISAT2 (v2.1.0), StringTie (v1.2.2), MAKER (v3.31.8), InterProScan (v5.16-55.0), RepeatMasker (v4.0.7), RepeatProteinMasker (v4.0.7), RepeatModeler (v1.0.11), Tandem Repeat Finder (v4.09), HMMER3, BLAST(v2.2.31);<br>Gene function annotation databases: KEGG, SwissProt, TrEMBL, NR, KOG54, GO, Repbase v21.12 library, Rfam database (Release 9.1);<br>Gene evolution analysis software: OrthoMCL (v1.4), CAFE (v2.1), ClusterProfiler (v4.0), MUSCLE (v3.8.31), RAxML, Ks values of gene pairs were calculated by TBtools (v1.0987), JCVI (MCSCAN python-version, v1.1.18);<br>Transcriptome analysis: Trimmomatic (v0.39), FastQC (v0.11.9), HISAT2 (v2.1.0), FeatureCounts (v1.6.3), DESeq2, TBtools(v1.0987);<br>Multiple sequence alignment: MEGAX (v10.2);<br>Phylogenetic analysis: MEGAX (v10.2) or iTOL(https://itol.embl.de/upload.cgi);<br>Quantification data analysis to make figures: Microsoft Excel;<br>Gene co-expression analysis : the heatmap was constructed by TBtools (v1.0987);<br>WGD events analysis : JCVI (MCSCAN python-version, v1.1.18), WGDi (v0.5.1);<br>Identification and visualization of gene families: The in-house analysis scripts have been deposited in Github (https://github.com/Zeyu-An/A.elata-genome-NEFU)<br>Editing of figures: Adobe Illustrator (v2020), ChemDraw (v14.0).

For manuscripts utilizing custom algorithms or software that are central to the research but not yet described in published literature, software must be made available to editors and reviewers. We strongly encourage code deposition in a community repository (e.g. GitHub). See the Nature Portfolio guidelines for submitting code & software for further information.

Data

Policy information about availability of data

All manuscripts must include a data availability statement. This statement should provide the following information, where applicable:

- Accession codes, unique identifiers, or web links for publicly available datasets
- A description of any restrictions on data availability
- For clinical datasets or third party data, please ensure that the statement adheres to our policy

The date supporting the findings of this work are available within the paper and its Supplementary Information files. The A. elata genome project has been deposited at the National Genomics Data Center (http:// bigd.big.ac.cn/) under BioProject no. PRJCA006215 (https://ngdc.cncb.ac.cn/search/?dbId=&q=PRJCA006215). Whole genome sequencing and RNA-seq data have been deposited in Genome Sequence Archive database (https://ngdc.cncb.ac.cn/gsa/) under accession nos. CRA004807 (https://ngdc.cncb.ac.cn/gsa/browse/CRA004807), CRA004817 (https://ngdc.cncb.ac.cn/gsa/browse/CRA004817), CRA004822 (https://ngdc.cncb.ac.cn/gsa/browse/CRA004822), CRA004827 (https://ngdc.cncb.ac.cn/gsa/browse/CRA004827) and CRA005648 (https://ngdc.cncb.ac.cn/gsa/browse/CRA005648). RNA-seq data has also been deposited at NCBI (https://www.ncbi.nlm.nih.gov/) under BioProject no. PRJNA755350 (https://www.ncbi.nlm.nih.gov/bioproject/?term=PRJNA755350). The A. elata genome sequence, annotation information, gene expression abundance of different tissues, and paralogous genes information also deposited at Dryad Digital Repository (https://doi.org/10.5061/dryad.69p8cz937). HMM files from Pfam (https://pfam.xfam.org/) were used to predict CYP450 (PF00067, https://pfam.xfam.org/family/PF00067/hmm), OSC (PF13249, https://pfam.xfam.org/family/PF13249/hmm and PF13243 https://pfam.xfam.org/family/PF13243/hmm), UDPGT (PF00201 https://pfam.xfam.org/family/PF00201/hmm) and CSL (PF03552 https://pfam.xfam.org/family/PF03552/hmm) gene families. The P. notoginseng and P. ginseng transcriptome data involved in this study were obtained from the SRA database (https://www.ncbi.nlm.nih.gov/sra/) under accession SRX2253710 (https://www.ncbi.nlm.nih.gov/sra/?term=SRX2253710) to SRX2253713 (https://www.ncbi.nlm.nih.gov/sra/?term=SRX2253713) and Ginseng Genome Database (http://ginsengdb.snu.ac.kr/index.php), respectively. Nucleotide sequence of the genes reported in this work have been deposited in NCBI (https://www.ncbi.nlm.nih.gov/) under accession OK094504, OK094505, OK094506, OK094507, OK094508, OK094509, OK094510, OK094511, OK094512, OK094513, OK094514, OK094515, OK094516 and OK094517. Source data are provided with this paper.

Field-specific reporting

Please select the one below that is the best fit for your research. If you are not sure, read the appropriate sections before making your selection.

[x] Life sciences [ ] Behavioural & social sciences [ ] Ecological, evolutionary & environmental sciences

For a reference copy of the document with all sections, see nature.com/documents/nr-reporting-summary-flat.pdf

Life sciences study design

All studies must disclose on these points even when the disclosure is negative.

Sample size	One sample of Aralia elata was used for genome sequencing; For RNA-seq three biological samples were used; For experiments determing metabolic contents three biological independent samples were used. All these samples were wild-type and were collected from the planting areas of A. elata
Data exclusions	For genome assembly, sequences with low-quality were excluded; We excluded the amino acids sequences that were lower than 300 aa for constructing the phylogenetic trees of P450s, CSLs, and UGTs.
Replication	For RNA-seq, three biological replicates were used; For metabolic analysis, three biological replicates were used.
Randomization	No method of randomization was used; No random sampling is required for genome sequencing, because the genome differences are very small within the wild population, thus any wild plant is allowed for genome sequencing; Biological replicates of RNA-seq were performed in separate experiments at different times to control for co-variates.
Blinding	Blinding is not applicable in our study because it does not involve subjects which receive different treatments. All experiments were done by analyzing data derived from different biological replicates directly.

Reporting for specific materials, systems and methods

We require information from authors about some types of materials, experimental systems and methods used in many studies. Here, indicate whether each material, system or method listed is relevant to your study. If you are not sure if a list item applies to your research, read the appropriate section before selecting a response.

Materials & experimental systems

n/a	Involved in the study
☒	☐ Antibodies
☒	☐ Eukaryotic cell lines
☒	☐ Palaeontology and archaeology
☒	☐ Animals and other organisms
☒	☐ Human research participants
☒	☐ Clinical data
☒	☐ Dual use research of concern

Methods

n/a	Involved in the study
☒	☐ ChIP-seq
☒	☐ Flow cytometry
☒	☐ MRI-based neuroimaging
